# Interaction networks of *Escherichia coli* replication proteins under different bacterial growth conditions

**DOI:** 10.1038/s41597-023-02710-1

**Published:** 2023-11-10

**Authors:** Joanna Morcinek-Orłowska, Beata Walter, Raphaël Forquet, Dominik Cysewski, Maxime Carlier, Michał Mozolewski, Sam Meyer, Monika Glinkowska

**Affiliations:** 1 Department of Bacterial Molecular Genetics, Faculty of Biology, University of Gdansk, Gdansk, 80-308 Poland; 2 Univ Lyon, Université Claude Bernard Lyon 1, INSA-Lyon, Lyon, CNRS, UMR5240 MAP, F-69622 France; 3 Mass Spectrometry Laboratory, Institute of Biochemistry and Biophysics, PAS, Warsaw 02-106, Warszawa, Poland

**Keywords:** Protein-protein interaction networks, Bacterial genetics

## Abstract

In this work we analyzed protein-protein interactions (PPIs) formed by *E. coli* replication proteins under three disparate bacterial growth conditions. The chosen conditions corresponded to fast exponential growth, slow exponential growth and growth cessation at the stationary phase. We performed affinity purification coupled with mass spectrometry (AP-MS) of chromosomally expressed proteins (DnaA, DnaB, Hda, SeqA, DiaA, DnaG, HolD, NrdB), tagged with sequential peptide affinity (SPA) tag. Composition of protein complexes was characterized using MaxQuant software. To filter out unspecific interactions, we employed double negative control system and we proposed qualitative and quantitative data analysis strategies that can facilitate hits identification in other AP-MS datasets. Our motivation to undertake this task was still insufficient understanding of molecular mechanisms coupling DNA replication to cellular growth. Previous works suggested that such control mechanisms could involve physical interactions of replication factors with metabolic or cell envelope proteins. However, the dynamic replication protein interaction network (PIN) obtained in this study can be used to characterize links between DNA replication and various cellular processes in other contexts.

## Background & Summary

Bacterial cell cycle consists of concurrent and interrelated processes: cell growth, chromosomal DNA replication and segregation culminated with cell division^1^. The essential processes of the cell cycle need to be coupled to nutrients availability to ensure safe and faithful transmission of genetic material to progeny cells. Under poor nutritional conditions or in slowly growing bacterial species, subsequent cell cycle events occur linearly, similar to eucaryotic cells. However, for fast-growing bacteria, in rich media, the time needed for synthesis of the full chromosomal copy exceeds the interval between subsequent divisions. To cope with that, at high growth rates all cell cycle stages occur simultaneously and, as a result, bacterial cells contain several replicating chromosomes at different replication stages. Nevertheless, irrespective of growth rate, DNA replication in *E. coli* and many other bacterial species starts at a defined cell volume/chromosomal origin ratio. All origins of replication (*oriC*), present in the cell at that time, fire simultaneously, once per division cycle^2–4^. However, molecular mechanism behind that size-dependent control remain uncertain.

The key component of the mentioned regulatory principles is certainly the DnaA protein. Its active, ATP-bound form accumulates at the entry to replication round and initiates a sequence of events at *oriC* leading to replication complex formation. Other crucial control elements encompass the DnaA protein regulators - DiaA, Hda and SeqA, of which the two former ones make a direct interaction with the replication initiator^5^.

Interestingly, multiple connections of replication control mechanisms to metabolism have been shown over the last years for bacteria with disparate cell cycle control, like *E. coli*, *Bacillus subtilis*, and *Caulobacter crescentus*^6–11^. It seems likely that those links can operate through physical interaction of the replication proteins with metabolites or metabolic enzymes and that those interactions may change under conditions supporting fast and slow growth rates. Moreover, a connection of DNA replication control to cell envelope synthesis has been suggested by showing that SeqA interacts with the outer membrane protein fraction and this association is temporally regulated through the cell cycle^12^. This increases potential DNA replication protein interaction network beyond metabolic enzymes. Exact molecular mechanisms underlying the links between replication and the mentioned processes remain largely unknown. Uncovering changes in the replication protein interaction networks (PIN) throughout different growth conditions may therefore foster identification of particular mechanisms employed by bacterial cells to coordinate the cell cycle with nutrient availability. Moreover, DNA-related processes, like transcription, DNA repair, and modification need to be coordinated with DNA replication. Those mechanisms are essential for genome stability from one generation to another and underscore its plasticity over evolutionary time scales.

Coordination of various processes in cells often takes form of direct protein-protein interactions between proteins belonging to distinct functional modules^13^. Therefore, the potential of replication factors dynamic PINs goes beyond information on growth rate-dependent control of replication initiation, and they can be used to study other aspects of bacterial chromosome biology.

**Fig. 1:**
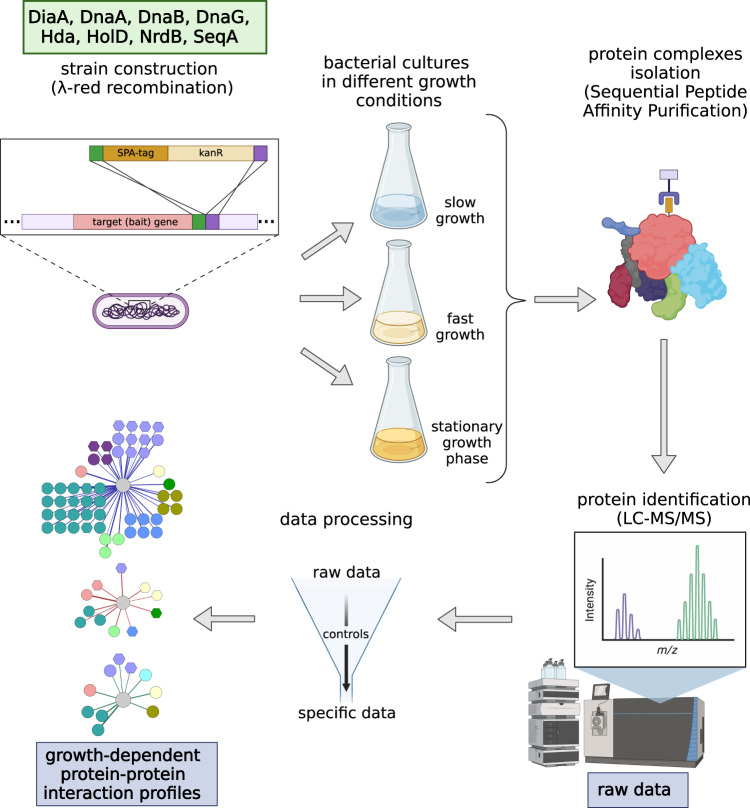
Experimental pipeline of the AP-MS protein-protein interaction screen. 8 different *E. coli* strains with SPA-tagged bait proteins were cultured in three disparate growth conditions and subjected to protein complexes isolation. Isolated proteins were identified using LC-MS/MS. As a result of two distinct data processing strategies we ended up with growth-dependent PINs of *E. coli* crucial replication proteins.

The aim of this work was to investigate the composition of protein complexes formed by the main factors involved in DNA replication in *E. coli* under three disparate growth conditions. We selected 8 bait replication proteins, including main DNA replication regulators (initiator protein DnaA, regulatory proteins DiaA, Hda and SeqA)^5^. Other baits encompassed replication complex components (DNA helicase DnaB, DNA primase DnaG, ψ subunit of DNA polymerase III HolD)^14^. We also included NrdB, a ribonuclotide reductase (RNR) subunit, the enzyme producing deoxyribonucleotides, experimentally suggested to associate with the replication complex and influencing its activity^15,16^. We have affinity-tagged the chosen bait genes at the native chromosomal positions in wild type (MG1655, K12 derivative) genetic strain, using sequential affinity purification (SPA) tag sequence^17,18^. This left them under control of their native promoters, to ensure near-endogenous levels of the replication proteins used as baits in our experiment. To assess protein-protein interactions of selected replication proteins, we used AP-MS according to the adjusted protocol published previously by Butland and coworkers^18,19^, followed by the identification of purified components using MaxQuant software environment^20^ (the whole experimental pipeline is depicted in Fig. 1). Replication machinery interactome was probed in the late exponential phase (OD_600_ ~ 0.6–1.0) during fast bacterial growth in rich, undefined medium (referred as LB log) and in defined synthetic medium, supporting slow growth rate, with acetate as a sole carbon source (referred as M9 0.2% ac ON); we also tested the PPI profile upon cell culture entry to stationary phase for LB-grown cultures (referred as LB ON). This way, we could subsequently compare changes within the replication proteins PPI network between fast and slow growth conditions, and after bacterial growth had ceased.

Large-scale analyses of PPI using SPA-tagged protein baits have been described before for well-known and orphan ORFs^19,21^ as well as cell envelope proteins^22^. However, they all were performed in standard laboratory conditions (rich medium, stationary growth phase) and with the use of DY330 (λ-Red recombination proficient) strain whose proteome might differ from wild type *E. coli*. Our dataset provides for the first time the insight of growth-dependent PIN dynamics of DNA replication proteins in wild-type *E. coli* strain. Moreover, we applied double-control system to filter out non-specific interactions with chromatography resins and SPA-tag and provided an easy-to-use, qualitative and quantitative data processing strategies that can be reused for other AP-MS datasets.

## Methods

### Strains, primers, and plasmids

List of all *E. coli* strains used in this study is presented in Table 1. Plasmids and primers are listed in Tables 2 and 3, respectively. All primers used to amplify linear DNA fragment used for λ-Red recombination-mediated SPA-tag integration contain constant sequences at their 3′ ends:

**Table 1:** *Escherichia coli* strains used in this study.

strain	genotype	source
MG1655	K-12 F– λ– ilvG– rfb-50 rph-1	Laboratory collection
MG1655 DiaA-SPA kanR	K-12 F– λ– ilvG– rfb-50 rph-1 diaA::diaA-SPA:kanR	This study
MG1655 DnaA-SPA kanR	K-12 F– λ– ilvG– rfb-50 rph-1 dnaA::dnaA-SPA:kanR	This study
MG1655 DnaB-SPA kanR	K-12 F– λ– ilvG– rfb-50 rph-1 dnaB::dnaB-SPA:kanR	This study
MG1655 DnaG-SPA kanR	K-12 F– λ– ilvG– rfb-50 rph-1 dnaG::dnaG-SPA:kanR	This study
MG1655 Hda-SPA kanR	K-12 F– λ– ilvG– rfb-50 rph-1 hda::hda-SPA:kanR	This study
MG1655 HolD-SPA kanR	K-12 F– λ– ilvG– rfb-50 rph-1 holD::holD-SPA:kanR	This study
MG1655 NrdB-SPA kanR	K-12 F– λ– ilvG– rfb-50 rph-1 nrdB::nrdB-SPA:kanR	This study
MG1655 SeqA-SPA kanR	K-12 F– λ– ilvG– rfb-50 rph-1 seqA::seqA-SPA:kanR	This study
MG1655 DiaA-SPA FRT	K-12 F– λ– ilvG– rfb-50 rph-1 diaA::diaA-SPA:frt	This study
MG1655 DnaA-SPA FRT	K-12 F– λ– ilvG– rfb-50 rph-1 dnaA::dnaA-SPA:frt	This study
MG1655 DnaB-SPA FRT	K-12 F– λ– ilvG– rfb-50 rph-1 dnaB::dnaB-SPA:frt	This study
MG1655 DnaG-SPA FRT	K-12 F– λ– ilvG– rfb-50 rph-1 dnaG::dnaG-SPA:frt	This study
MG1655 Hda-SPA FRT	K-12 F– λ– ilvG– rfb-50 rph-1 hda::hda-SPA:frt	This study
MG1655 HolD-SPA FRT	K-12 F– λ– ilvG– rfb-50 rph-1 holD::holD-SPA:frt	This study
MG1655 NrdB-SPA FRT	K-12 F– λ– ilvG– rfb-50 rph-1 nrdB::nrdB-SPA:frt	This study
MG1655 SeqA-SPA FRT	K-12 F– λ– ilvG– rfb-50 rph-1 seqA::seqA-SPA:frt	This study
DY330 DnaA-SPA kanR	W3110 ∆lacU169 gal490 λCI857 ∆(cro-bioA) dnaA::dnaA-SPA:kanR	GE Healthcare Dharmacon
DH5a	*fhuA2 lac(del)U169 phoA glnV44 Φ80’ lacZ(del)M15 gyrA96 recA1 relA1 endA1 thi-1 hsdR17*	Laboratory collection
Rosetta pLysS	F^–^ *ompT gal dcm lon hsdS*_*B*_(*r*_*B*_^–^*m*_*B*_^–^) λ(DE3 [*lac lacUV5*-*T7p07 ind1 sam7 nin5*]) [*malB*^+^]_K-12_(λ^S^) pLysSRARE[*T7p20 ileX argU thrU tyrU glyT thrT argW metTleuW proL ori*_p15A_](Cm^R^)	Laboratory collection

**Table 2:** Plasmids used in this study.

plasmid	description	Source/reference
pUC19-pIVSK	pUC19 backbone with cloned mVenus-SPA sequence under the control of constitutive promoter placI.	This work
pKD46	Temperature-sensitive Red recombinase expression plasmid.	^24^
pCP20	Temperature-sensitive plasmid containing FLP gene to remove FRT-flanked antibiotic resistance cassette.	^24^
pRK793	Expression vector containing the gene encoding TEV protease. The induction of pRK793 with IPTG produces an MBP fusion protein that self-cleaves *in vivo* to generate a soluble His6-TEV protease.	^27^

**Table 3:** Primers used in this study.

Primer name	sequence	description
diaA-SPAkan F	ATTGCCTGTGCGATCTGATCGATAACACGCTTTTCCCTCACCAGGATGAT	SPA-tag integration
diaA-SPAkan R	AGCGCGGAAATAAGGACTGCGATTGGCGATAATGCCTTCATGTATTCTCC	
dnaA-SPAkan F	GCCACGATATCAAAGAAGATTTTTCAAATTTAATCAGAACATTGTCATCG	
dnaA-SPAkan R	GTTGTAGCGGTTTTAATAAATGCTCACGTTCTACGGTAAATTTCATAGGT	
dnaB-SPAkan F	GTCAATGGTCGCGCTTCGACAACTATGCGGGGCCGCAGTACGACGACGAA	
dnaB-SPAkan R	GTGTTCCTTGATAAGTGTTTGCTTTAATTACCTAATTCATAAAATAATTA	
dnaG-SPAkan F	ACGAAGAACGCCTGGAGCTCTGGACATTAAACCAGGAGCTGGCGAAAAAG	
dnaG-SPAkan R	TGCGGCTGTCGGGGGCTTCCCGATCGCTCTTCGGCACTTAAGCCGTTAAA	
holD-SPAkan F	TATGGCAACAAATTTGCACATATGAACACGATTTCTTCCCTCGAAACGAC	
holD-SPAkan R	TCCACGGAAAGGCGTGGGCGCGTTGTTCAATGTGGTAAGCCGCCGGTAAA	
hda-SPAkan F	CCGCGCAACGTAAGCTGACCATTCCGTTTGTGAAAGAAATTCTGAAGTTG	
hda-SPAkan R	GCGTAGTTCGGATAAGGCGTTCGCGCCGCATCCGACAATAAACACCTTAT	
nrdB-SPAkan F	GGCAGATTGACTCGGAAGTGGACACCGACGATTTGAGTAACTTCCAGCTC	
nrdB-SPAkan R	ATCCTGGCACAGCAGTTGTGTGCCAGTGATGCGCAGGGTAACGCGGGCCA	
seqA-SPAkan F	AGTCGATGCAATTCCCGGCGGAATTGATTGAGAAGGTTTGCGGAACTATC	
seqA-SPAkan R	GGCCTGCACGATTGTGGATTGCCATTGCTTTGTCCTTTGTCTGCAACGTT	
S diaA F	TTGTTAGGGCCACAGGATGT	Recombinants screening
S diaA R	GACACTGCGTGGGTCAGTT	
S dnaA F	CTTCATGCCTGCCGTAAGAT	
S dnaA R	CGTACCGTCAGCAACCTGTA	
S dnaB F	AGGCATCGCGGAAATTATTA	
S dnaB R	ACCACCGCAACCATTTTACT	
S dnaG F	GAGCAAACCTTCACCGACTC	
S dnaG R	GCTGAAATCCAACGGTTGTT	
S holD F	ACAGTTGGCGGTTGGGTACT	
S holD R	ATTTTGCCGTTTTGCGTTA	
S hda F	TTTGAACTGCCGGAAGATGT	
S hda R	CCATCGCTAGTTGAAGCACA	
S nrdB F	GATCCCGTGGATCAACACTT	
S nrdB R	TCGCGACACTGGTACTCAAC	
S seqA F	GATGAACAAACGCTGCTGAA	
S seqA R	GTCAGTTGGGCGACGTTAAT	

F: 5′-overhang-TCCATGGAAAAGAGAAG-3′

R:5′-overhang- CATATGAATATCCTCCTTAG-3′

Thus, only variable 5′-terminal sequences (overhangs) of the primers described as ‘integration’ primers are presented in Table 3.

Cloning of pUC19-pIVSK was performed using restriction-free cloning procedure, as described in^23^.

### Bacterial cultures and media

LB Lennox medium (0.5% yeast extract, 1% tryptone, 0.5% NaCl) and M9 acetate medium (1x M9 minimal salts, 2 mM MgSO4, 0.1 mM CaCl2, 0.05% thiamine, 25 μg/ml uridine, 0.2% sodium acetate) components were purchased from either Roth GmbH or Sigma-Aldrich. All overnight cultures were grown in LB Lennox medium. If needed, ampicilin (Sigma-Aldrich) or kanamycin (Sigma-Aldrich) were added to the final concentration of 50 μg/ml.

Large-scale bacterial cultures for protein complexes purification were prepared in 2 liters of medium and inoculated with 20 ml of an overnight culture. Large-scale cultures were grown at 37 °C to late exponential phase (OD_600_ = 0.6–1.0) (in the case of LB log and M9 acetate) or to stationary phase.

### Construction of SPA-tagged *E. coli* strains

**Fig. 2:**
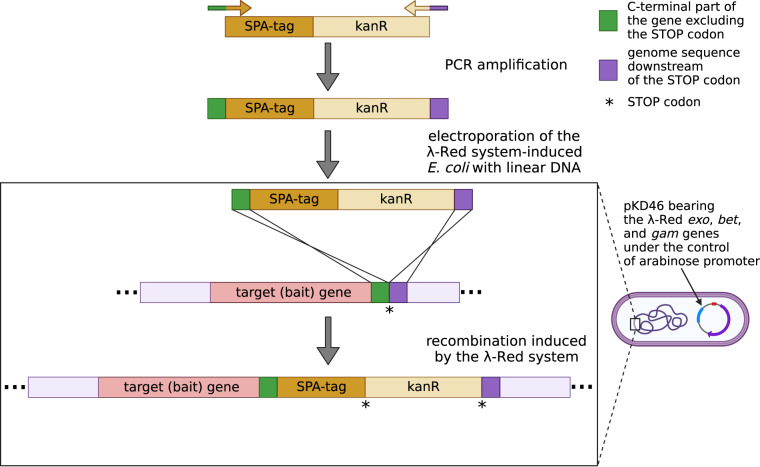
Strain construction scheme for strains used in this study. DNA fragment with SPA-tag sequence and kanamycin resistance cassette was PCR-amplified using primers with overhangs specific to the chromosome integration site and electroporated into *E. coli* cells where the λ-Red system recombination genes were induced from pKD46 plasmid.

All strains used for isolation of bacterial protein complexes were based on MG1655 genetic background (Table 1). SPA-tagged strains were constructed by one-step integration of linear DNA fragment containing SPA-tag sequence and kanamycin resistance cassette using λ-Red recombination method^24^ (Fig. 2). DNA fragments for integration were PCR-amplified with Phusion Flash DNA polymerase (Thermo Scientific) using genomic DNA of commercial, DY330 SPA-tagged strain as template. Primers used for PCR amplification consist of 20nt sequences specific to SPA-tag-kan^R^ and 50nt 5′-overhangs homologous to the chromosomal regions on either side of the integration site. PCR products were column-purified, eluted with ultra-pure distilled water and used for electroporation. Electrocompetent cells were prepared of MG1655 strain harboring pKD46 plasmid, expressing the λ-Red recombination proteins under the control of arabinose promoter. The cells were grown in LB + amp at 30 °C to an OD_600_ of ~0.6, subsequently induced with 0.15% L-arabinose and grown for additional hour at 37 °C. Then the cells were pelleted and subjected to three rounds of washing and pelleting, twice with distilled water and once with 10% glycerol, both ice-cold. Finally, cells were concentrated 100-fold in 10% glycerol. For each transformation, 80 μl of competent cells was mixed with ~1 μg of PCR product. Electroporation was done with Eppendorf Eporator using 2500 V and 0,1 cm chambers. Electroporated cells were added to 1 ml LB, incubated for 3 h at 37 °C and spread onto LB plates with proper antibiotic. Positive transformants were PCR-verified, sequenced and subjected to FRT-FLP recombination to eliminate antibiotic resistance cassette, as described in^24^.

### Chromosome copy number assessment after replication runout

Wild type MG1655 strain grown in different media was subjected to rifampicin-cephalexin treatment (the whole procedure is described in details in^25^) at early exponential phase (OD_600_ ~ 0.15) to stop the following replication initiation round and cell division. As a result, bacterial cell contains the number of chromosome copies corresponding to the number of actively replicating origins at the moment of antibiotics treatment. The chromosome copy number was measured after DNA staining with Sytox green dye using BD FACS Calibur flow cytometer. We used four different media previously reported to ensure different growth rates^26^, including minimal medium with acetate and LB medium (additionally supplemented with glucose), the conditions used in PPI screen presented in this work.

### TEV protease expression and purification

TEV protease was overproduced in *E. coli* Rosetta (DE3) pLysS cells from pRK793 plasmid^27^ in the form of an MBP fusion protein that cleaves itself *in vivo* to yield a TEV protease catalytic domain with an N-terminal His-tag and a C-terminal polyarginine tag. Overexpression was performed for 18 hours at 20 °C after addition of 1 mM isopropyl β-D-1-thiogalactopyranoside (IPTG). Cells were harvested, lysed by sonication in buffer A (50 mM Tris-HCl pH 8.0, 400 mM NaCl, 5% glycerol, 5 mM β-mercaptoetanol, 15 mM imidazole) supplemented with 1.5 mM phenylmethanesulfonylfluoride (PMSF) and 1 tablet of Pierce™ Protease Inhibitors (Thermo Scientific, A32965). The lysate was cleared by centrifugation. The protein was purified from the soluble fraction of the lysate by Ni-affinity chromatography on a 5 ml HisTrap column (GE Healthcare) equilibrated with buffer A. The column was washed with buffer B (50 mM Tris-HCl pH 8.0, 1 M NaCl, 5% glycerol, 5 mM β-mercaptoetanol, 15 mM imidazole). Subsequently, the protein was eluted with buffer C (50 mM Tris-HCl pH 8.0, 400 mM NaCl, 5% glycerol, 5 mM β-mercaptoetanol, 500 mM imidazole) and dialyzed into buffer D (50 mM Tris-HCl pH 8.0, 200 mM NaCl, 10% glycerol, 2 mM β-mercaptoetanol). Protein concentration was estimated using NanoDrop Spectrophotometer (Thermo Scientific) at 280 nm and aliquots of purified protein were frozen in liquid nitrogen and stored at −70 °C.

### Protein complexes purification

**Fig. 3:**
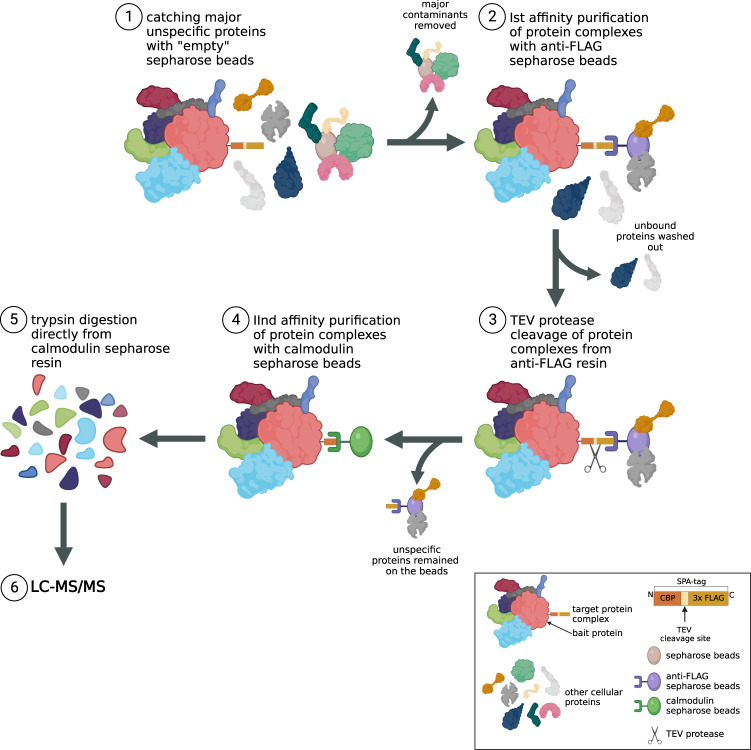
Sequential Peptide Affinity (SPA) purification procedure. Soluble fraction of proteins was incubated with sepharose 4B resin to get rid of major contaminants unspecifically interacting with the beads (step 1). Protein complexes were subsequently immobilized on the anti-FLAG sepharose beads (step 2) and cleaved from the resin using TEV protease (step 3). Next, second affinity purification round with calmodulin sepharose beads was perfomed (step 4) followed by trypsin digestion directly from the resin (step 5) right before LC-MS/MS (step 6).

Isolation of SPA-tagged bacterial protein complexes was performed according to the detailed protocol published by Babu and coworkers^18^, with several modifications, according to the scheme presented in Fig. 3. Briefly, cell pellets, harvested by centrifugation, were resuspended in 20–40 ml of sonication buffer (20 mM Tris pH 7.9, 100 mM NaCl, 0.2 mM EDTA, 10% glycerol, 0.1 mM DTT). The cell slurry was supplemented with 1 tablet of Pierce™ Protease Inhibitors (Thermo Scientific, A32965) per 50 ml of buffer and lysed by sonication. Cell debris was removed by centrifugation at 18000 rpm for 45 min. Cleared lysate was incubated with 50–75 U of Viscolase nuclease (A&A Biotechnology) for 30 min on ice. After degradation of nucleic acids, Triton X-100 was added to the lysate to the final concentration of 0.1%. The lysate was incubated with 250 μl of Sepharose® 4B-200 (Sigma-Aldrich), pretreated by washing with AFC buffer (10 mM Tris pH 7.9, 100 mM NaCl, 0.1% Triton X-100), for 1 h at 4 °C with gentle rotation (Fig. 3, step 1). This step was performed to decrease the amount of proteins sticking unspecifically to the resin. The beads were separated from the lysate, which was subsequently incubated with anti-FLAG Sepharose (Biotool, B23102), pretreated by washing with AFC buffer, for 3 h at 4 °C with gentle rotation (Fig. 3, step 2). The beads were subsequently collected by centrifugation at 4000 rpm for 15 min and transferred into mini-spin column. Beads were washed on column three times with 250 µl of AFC buffer and twice with 250 µl of TEV cleavage buffer (50 mM Tris pH 7.9, 100 mM NaCl, 0.1% Triton X-100) to remove unbound proteins. 8 μl of in-house purified TEV protease (conc. ~5 mg/ml) in 250 μl of TEV cleavage buffer was added to the closed column and incubated overnight at 4 °C (Fig. 3, step 3). The next day supernatant containing cleaved proteins was collected, mixed with CaCl_2_ to the final concentration of 1.5 mM and incubated for 3 h at 4 °C with gentle rotation with Calmodulin Sepharose (GE Healthcare, 17-0529-01), pretreated by washing with CBB buffer (10 mM Tris pH 7.9, 100 mM NaCl, 2 mM CaCl2, 0.1% Triton X-100) (Fig. 3, step 4). The protein-bound beads were transferred into a new mini-spin column, washed twice with 250 µl of CBB buffer and three times with 250 µl of CWB buffer (10 mM Tris pH 7.9, 100 mM NaCl, 0.1 mM CaCl2). Dried beads were stored at −20 °C and subjected directly to trypsin digestion prior to Liquid Chromatography coupled to tandem Mass Spectrometry (LC-MS/MS) (Fig. 3, steps 5 and 6). Each sample was prepared in three biological replicates.

### Identification of proteins by LC-MS/MS

Dried beads were suspended in 50 μl of 100 mM NH4HCO3 and reduced with TCEP on a shaker at RT, alkylated with iodoacetamide in darkness for 45 min at RT on the shaker and digested overnight with 10 ng/μl trypsin. Digestion was stopped with 5%TFA to a final concentration of 0.1%, acetonitrile was added to a final concentration of 2%.

The resulting peptide mixtures were separated and measured at an online LC-MSMS setup. LC (Waters Accuity) RP-18 pre-columns (Waters), nano-UPLC RP-18 column (internal diameter: 75 μM, 250 mm long, Waters) using an acetonitrile gradient (2%–35% ACN in 180 min) in the presence of 0.1% trifluoroacetic acid at a flow rate of 250 nl/min. The column outlet was directly coupled to the ion source of an Orbitrap Elite mass spectrometer (Thermo Scientific). Measurements were conducted in positive polarity mode, with the capillary voltage set to 2.5 kV. The mass spectrometer was operated in a data-dependent mode. Higher-energy Collisional Dissociation (HCD) fragmentation was applied. Up to 10 MS/MS events were allowed per MS scan. Resolution of MS 30 000, MSMS 15 000, MS m/z range 300–2000, isolation width 3, normalized collision energy 32.0. Three blank washing runs were done between each sample to ensure the absence of cross-contamination from preceding samples. Analysis was performed at the Laboratory of Mass Spectrometry (IBB PAS, Warsaw). Data were analyzed using MaxQuant 1.6.3.4, referenced to *E. coli* proteome from UniProt database downloaded on 25.05.2020, 4391 entries. In total, 1600 proteins were identified (FDR 1%). The error ranges for the first and main searches were 20 ppm and 6 ppm, respectively, with 2 missed cleavages. Carbamidomethylation of cysteines was set as a fixed modification, and oxidation and protein N-terminal acetylation were selected as variable modifications for database searching. The minimum peptide length was set at 7 aa. Both peptide and protein identifications were filtered at a 1% false discovery rate and were thus not dependent on the peptide score. Enzyme specificity was set to trypsin, allowing cleavage of N-terminal proline. A ‘common contaminants’ database (incorporated in MaxQuant software) containing commonly occurring contaminations (keratins, trypsin etc.) was employed during MS runs.

### Protein complexes - data processing

To determine unspecific interactants within our PPI screen, we included two types of control samples in our experiment. First control was based on untagged *E. coli* strain MG1655 (laboratory wild type strain), that is the genetic background of all the strains used in this study. It shows the proteins that unspecifically interact with the resins used during protein complexes isolation. Second control contains heterologous SPA-tagged protein (we used fluorescent protein mVenus) whose gene, under the control of constitutive lacI promoter, was delivered to MG1655 *E. coli* strain on high-copy plasmid pUC19-pIVSK. This control, in turn, enables to determine which proteins might unspecifically interact with the SPA-tag itself. Both control samples were subjected to the same purification procedure as the experimental ones, and were also prepared in three biological replicates.

**Fig. 4:**
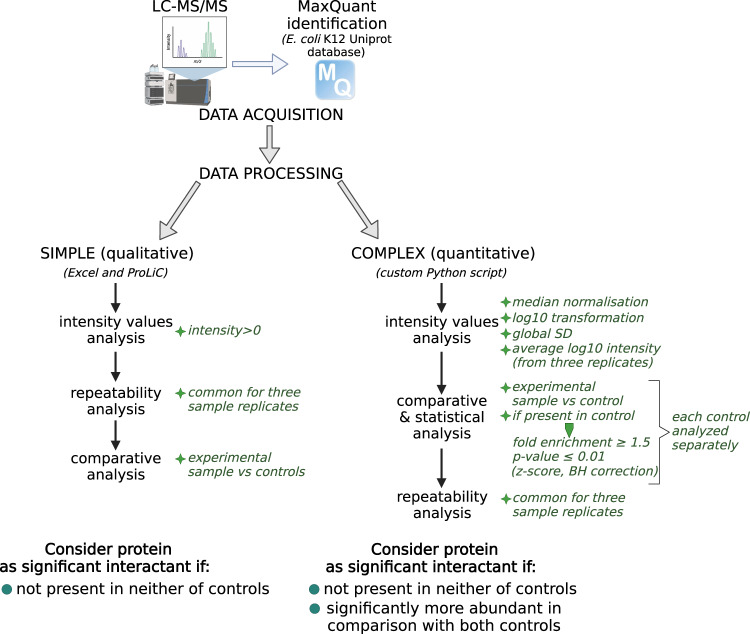
Simple (qualitative) and complex (quantitative) processing strategies of raw MaxQuant data. Both strategies refer to the double control system, but the qualitative strategy is based solely on the presence/absence of interactant in both controls, whereas in the quantitative strategy the intensity values are compared between experimental and control samples, and statistical test is performed.

After MaxQuant analysis, we processed the data in two distinct ways. Both processing strategies aim to filter out non-specific interactants, but the simple (qualitative) processing assumes that any protein that appears in at least one type of control should be rejected, whereas the complex (quantitative) strategy relies on intensity values and consider protein interactant as specific not only if absent in two control samples, but also if significantly more abundant in experimental than in control sample. The simple strategy uses mainly Protein List Comparator (ProLiC) in accession-based mode^28^. The complex data processing strategy, in turn, was done using custom Python script (version 3.7.6). Overall scheme of data processing is presented in Fig. 4. The detailed, consecutive steps in both types of processing are described below.

Simple (qualitative) data processing:

1. Sort intensity values from highest to lowest and keep only proteins with intensity >0
2. Prepare ProLiC input file of each sample replicate where the first column is protein accession ID, the second column is biomolecule type (‘protein’), and the subsequent columns are gene name, protein description and intensity value.
3. Compare three replicates of each experimental and control sample using ProLiC in accession-based mode. Keep only proteins common for all three sample replicates.
4. For each experimental and control sample of given growth condition, prepare ProLiC input file containing common-three replicates’ proteins as described in step 2.
5. Compare each experimental sample from given growth condition with control 1 and control 2 using ProLiC in accession-based mode.
6. Consider protein as significant interactant if it appears only in the experimental samples and is absent in all replicates of both controls.

Complex (quantitative) data processing:

1. Normalize each protein intensity value per median intensity in the sample to reduce inter-sample variability. If intensity = 0 (protein absent in the sample replicate), replace the value with 1 (the value that gives 0 after log10 transformation).
2. Apply log10 transformation to the normalized intensity values (referred as ‘intensity’ in the next processing steps).
3. Calculate global variance and global standard deviation from all intensity values among all samples from given growth condition.
4. For each experimental and control sample, calculate average intensity value from three replicates of given growth condition (referred as ‘av_intensity’ in the next processing steps).
5. Compare each av_intensity value of experimental sample to the corresponding value of control 1. If the control av_intensity equals 0, consider protein as significant in relative to control 1 (protein absent in control 1). If not, go to the next step.
6. Compute the av_intensity log2 magnitude ratio between experimental and control sample. Keep only proteins with difference between control and experimental sample ≥ 1.5.
7. Assess the significance of the fold enrichment with a one-way z-score using global standard deviation. Adjust obtained p-value using Benjamini-Hochberg correction for multiple-testing. Keep only proteins with adjusted-pvalue ≤ 0.01.
8. Repeat steps 5–7 with control 2.
9. Check if proteins that meet the previous conditions are present in all three replicates of experimental sample.
10. Consider protein as significant interactant if it appears in all three experimental sample replicates and if it is absent or significantly enriched relative to both controls.

### Data visualization and figures

Venn diagrams were made with a free online tool: http://bioinformatics.psb.ugent.be/webtools/Venn/. Protein interaction networks were analyzed and visualized using Cytoscape ver. 3.8.2. Volcano plots were made using VolcanoseR tool: https://goedhart.shinyapps.io/VolcaNoseR/. All Figures from the manuscript were prepared using https://biorender.com.

## Data Records

**Fig. 5:**
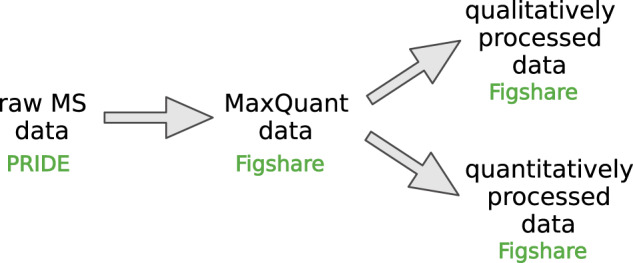
A scheme depicting organization of raw MS data and processed data on PPI interactions between different files.

The organization of our deposited data is depicted in Fig. 5. Briefly, as a result of MaxQuant identification of raw MS data, the raw MaxQuant protein lists were generated; these files served as input files to perform qualitative and quantitative data processing strategy. Raw MS data were deposited in Pride Repository under an entry PXD030113^29^. MaxQuant data (input files for our processing strategy), as well as quantitatively and qualitatively processed data, were deposited as protein lists in Excel files at Figshare Repository^30–32^. The coding of data samples included in this work is presented in Table 4 (short version containing MaxQuant datafiles’ names) and Supplementary Table 1 (full version including corresponding raw MS datafiles’ names).

**Table 4:** MaxQuant data sample coding.

sample type (experimental(E)/control(C)): bait protein	growth condition		
	LB log	LB O/N	M9 0.2% ac O/N
E:**DiaA**	E1; I4; X6	I5; P1; X7	I6; T3; X5
E:**DnaA**	F2; U6; U7	F1; P2; X12	E3; I7; X11
E:**DnaB**	G4; W3; W4	G6; W5; W6	G2; W1; W2
E:**DnaG**	H1; J5; U9	C7; F6; H2	F7; H3; X15
E:**Hda**	C5; U8; X9	C6; P3; X10	I3; T4; X8
E:**HolD**	U1; U2; U3	F8; U11; X4	X1; X2; X3
E:**NrdB**	W7; W8; W9	F10; J4; U13	F9; J3; T10
E:**SeqA**	F4; X13; X14	F3; P4; U12	F5; T1; T2
C: MG1655 **(TYPE 1 CONTROL**)	T7; T8; T9	P5; U10; X17	T5; T6; X16
C:MG1655 (placI)mVenus-SPA-pUC19 **(TYPE 2 CONTROL)**	S1; S2; S3	O9; R4; R5	R1; R2; R3

Processed data are deposited as excel files containing protein lists along with the basic protein information such as ID, gene and protein name. Both data processing strategies contains 8 Excel files (one separate file for each bait protein), each with 3 sheets containing identified interactants for different growth conditions. Datafiles of each processing strategy are provided with metadata file, with the meaning of every column and a brief instruction about the table contents. Depending on the processing strategy, additional parameters included in the datafiles are different. In case of complex (quantitative) data processing^31^, all calculations on the intensity value are present in the datafile as well as the statistical test’s p-value and difference between the samples and both controls. Even though we did not consider intensity values during qualitative processing strategy^32^, we have decided to leave the normalized intensity value also in the qualitatively processed datafiles to give the user an opportunity to sort and compare the data between samples if needed.

All Figures, tables and Appendix files were also uploaded to Figshare Repository^33^

## Technical Validation

### Experimental setup and data processing strategies

**Fig. 6:**
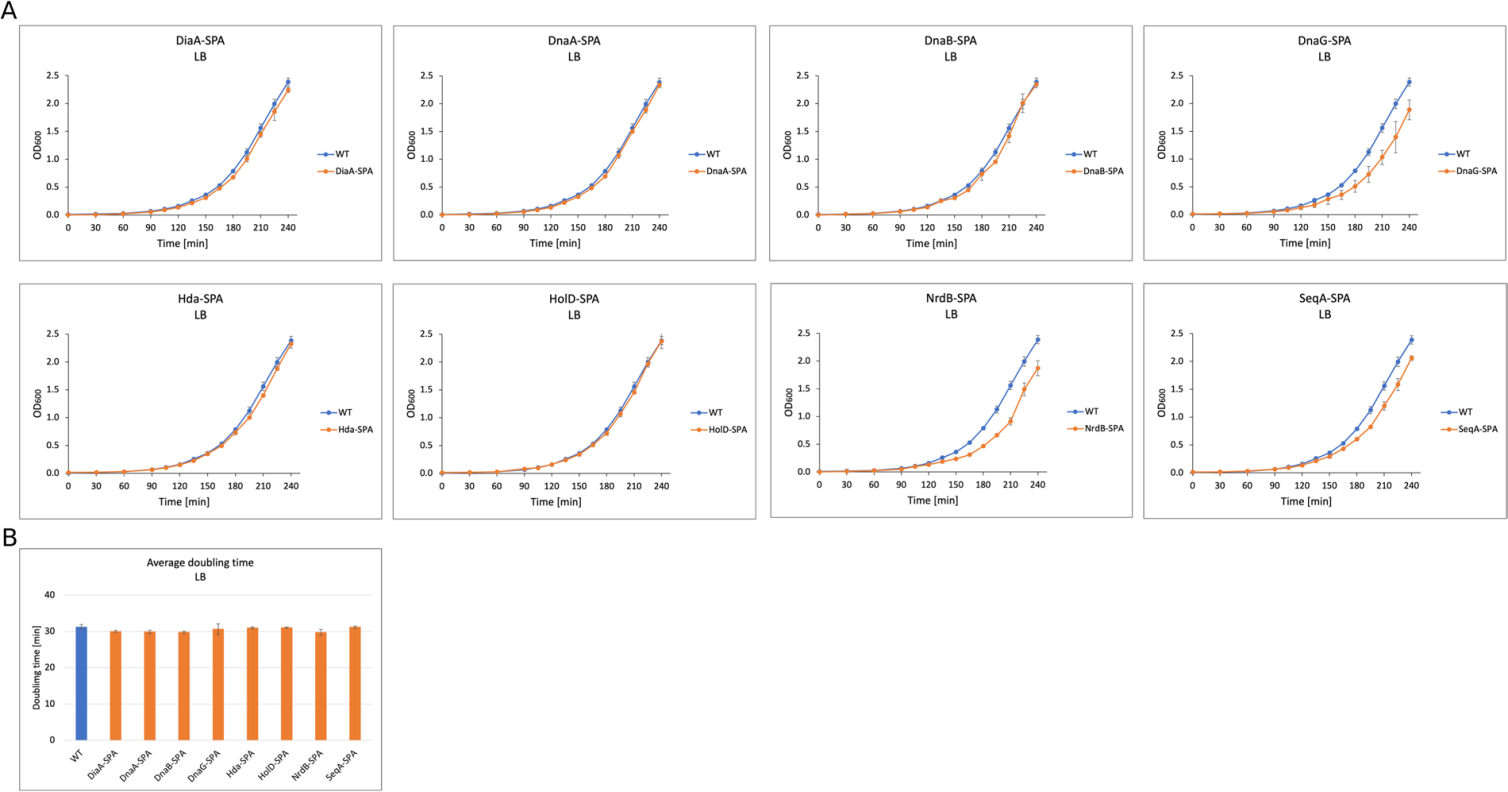
Growth curves and generation time comparison for the wild type and SPA-tagged strains used in this study growing in LB medium.

**Fig. 7:**
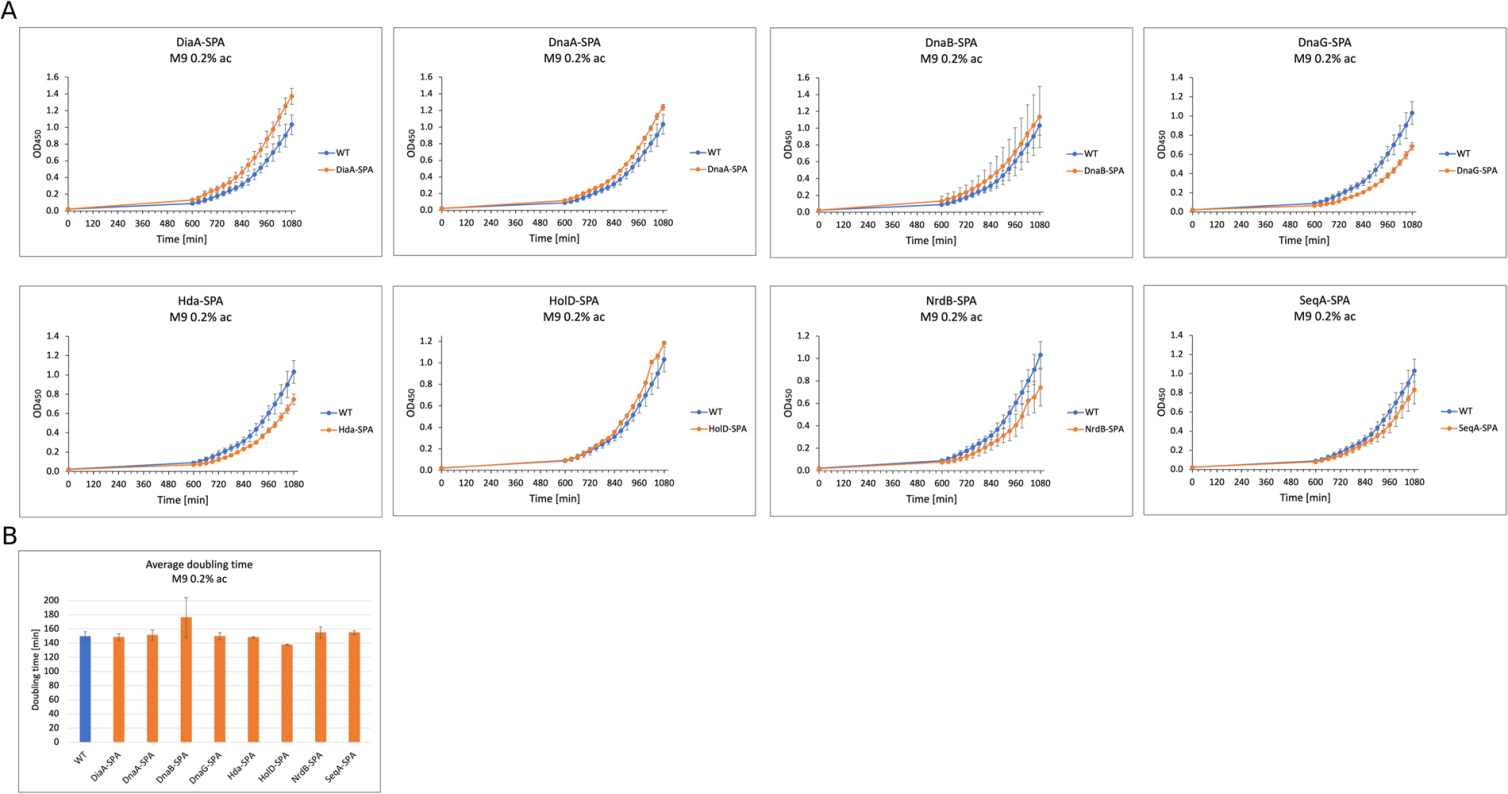
Growth curves and generation time comparison for the wild type and SPA-tagged strains used in this study growing in M9 + 0.2% acetate medium.

To assess if growth conditions chosen for the PPI screen indeed ensure different growth rates and replication frequency, changes in the bacterial cell cycle were confirmed using flow cytometry measurement of chromosome copy number after replication run-out. As expected, bacterial cells grown in LB medium exhibit overlapping replication cycles indicated by populations of 8 and 16 chromosome copies. In turn, growth in minimal medium supplemented with sodium acetate resulted in populations containing 1 or 2 copies of chromosomes, that is non-overlapping replication rounds (Supplementary Figure 1)^26^. Additionally, we tested growth rates of all constructed SPA-tagged strains in both microbial media used for our PPI screen and found no considerable differences from the wild-type strain, indicating that addition of the SPA-tag did not interfere considerably with strain physiology (Figs. 6 and 7 and Appendix files 1 and 2)^33^.

Affinity-directed proteomics is often strongly biased with false-positive results. Proteins may interact unspecifically with affinity tags and chromatography resins used during isolation process which obscures subsequent identification of true interactants. This issue is partially solved during sequential purification procedure: pre-incubation with empty resin and cutting protein complexes after the first affinity binding with sequence-specific TEV protease should decrease the amount of unspecific proteins remained in subsequent steps (see Fig. 3). We also tested that the TEV protease batch used cleaved the bulk bait protein off the anti-FLAG resin (Supplementary Figure 2).

However, the level of unspecific interactants identified after MS is still significantly high, therefore, efficient system to separate the wheat from the chaff needs to be developed. To tackle this problem, we performed two types of control experiments. The first involved an untagged wild type strain (MG1655 *E. coli* strain – genetic background in our experiments) and the second - the wild type strain expressing a SPA-tagged fluorescent Venus protein from plasmid. Both types of control samples (in triplicates) were grown under identical conditions to those used for the strains expressing the tagged bait proteins and were subsequently subjected to identical purification procedure. In the first case, the control experiment enabled correction for proteins attaching unspecifically to the resins, in the second – for proteins binding to a random SPA-tagged protein or SPA-tag itself. The use of the two control types delimits abundance range of a protein that binds unspecifically, dependent on resin occupancy by a bait protein. Specifically, we made a presumption that the level of proteins binding unspecifically to the resin will be lower when the amount of bait protein and its interactants is high and thus, the resin beads are more occupied. The differences between resin occupancy among different bait proteins may result from different native protein expression levels as well as various tag surface exposition on the natively folded proteins.

**Fig. 8:**
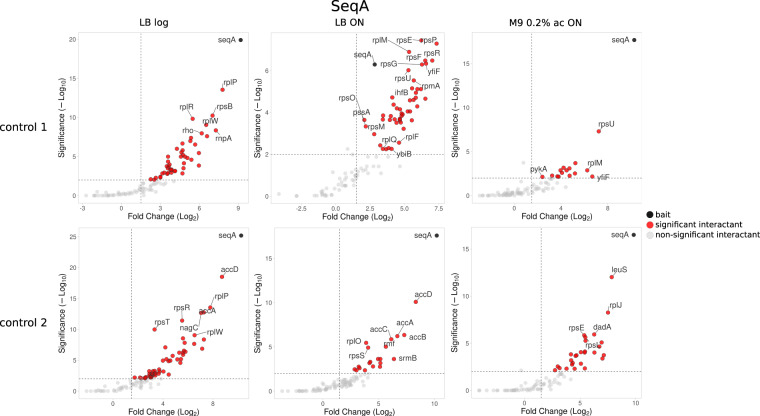
Volcano plots depicting enrichment and statistical significance of uncovered interactions for one of the bait proteins - SeqA.

Using very restrictive criteria for hits identification, the presence of a protein in at least one of the controls described above should disqualify it as a true-positive interactant. However, considering that MS is a sensitive technique, not every protein forming an interaction with the resins or SPA-tag itself should be automatically accounted for as a false-positive as long as it has been identified in significantly higher amount in experimental sample containing tagged bait protein. Therefore, we present the data processed with the same control samples, but using two different strategies, described in details in Methods section. Simple (qualitative) strategy is based only on the presence of protein hits in control samples, regardless of their intensity values, whereas complex (quantitative) strategy allows to calculate protein intensity enrichment value and assess its statistical significance (Fig. 4). We also validated that the bait protein was in each case the most highly enriched one (Fig. 8, Supplementary Fig. 3A-G).

### Data reproducibility

**Fig. 9:**
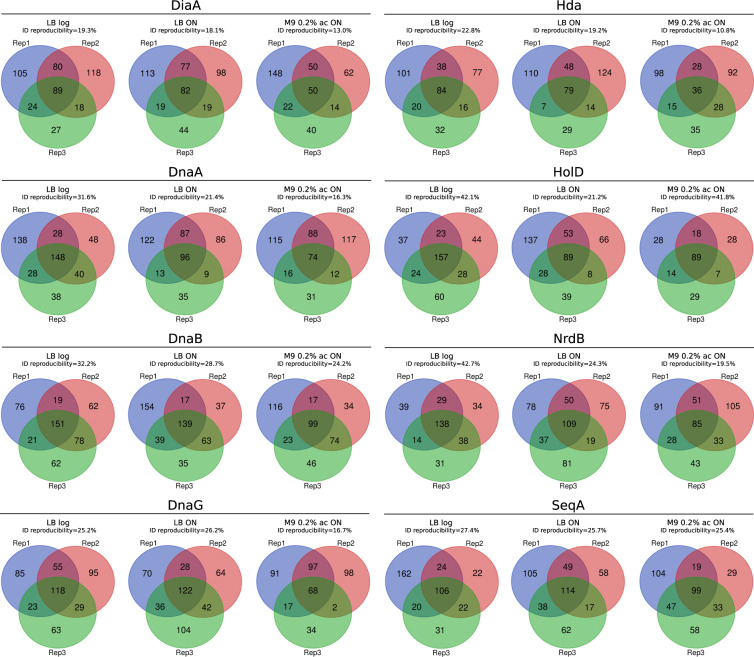
Venn diagrams presenting the protein IDs overlap between three experimental replicates for each bait protein.

As a result of MaxQuant analysis (referenced to *E. coli* proteome from UniProt database containing 4391 different protein IDs), 1596 different protein IDs were identified within all the samples used for searching (FDR 1%)^29^. Depending on the growth condition, the average numbers of identified protein IDs in the samples (including experimental and control ones) were as follows: 250 (LB log), 212 (M9 0.2% ac ON) and 248 (LB ON). The bait protein intensity across different growth conditions was similar (Supplementary Figure 4). The data reproducibility between three sample replicates is presented on Venn diagrams (Fig. 9 and Appendix file 3)^33^. Protein IDs that appeared in all three replicates constituted 10.8–42.7% of all different protein IDs for a given bait. For downstream data processing we included only proteins that appeared in all three sample replicates. In qualitative processing strategy^32^, we compared common-three replicates protein set of experimental and control samples. In turn, in quantitative strategy^31^, we performed statistic tests for every common-three-replicates protein hit that appeared in at least one replicate of the control samples (Fig. 4). Statistic tests were performed separately for each control, and we further considered as hits only the interactions that were statistically significant with respect to both control types as well as preys that did not appear in any of the controls.

**Fig. 10:**
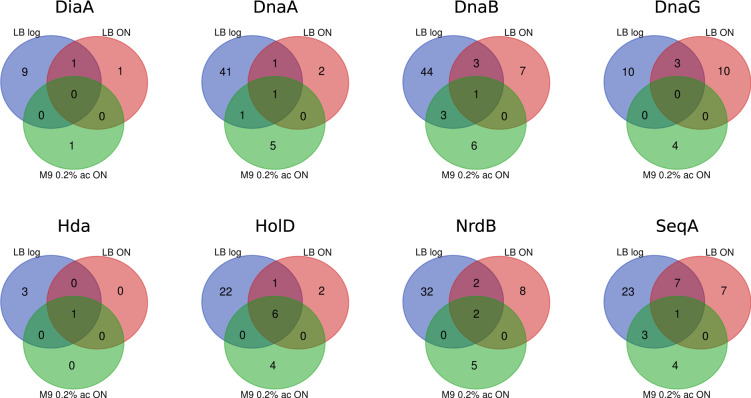
Venn diagrams presenting the protein IDs overlap between three growth conditions for each bait protein (quantitative data processing).

**Fig. 11:**
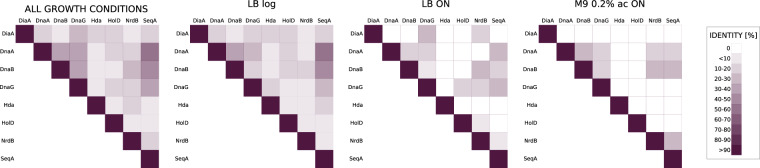
Pairwise comparison of uncovered interaction profiles of 8 baits used in this work (quantitative data processing). Similarity matrices were made based on comparison of interaction profiles between each of 8 bait proteins used in PPI screen (‘each with each’ comparison). Percent of the same interactants between compared baits was calculated and this value was weighted-averaged to the number of all interactants identified in our PPI screen.

Importantly, the analysis of quantitatively processed data revealed that the set of interactants for every bait used in our screen changed drastically with growth conditions (Fig. 10). In general, the highest number of significant interactions was observed in samples obtained from bacteria during their exponential growth in rich medium, whereas the smallest – in samples grown in minimal medium with acetate. This difference in significant prey number can only partially be attributed to bait abundance in samples from different growth conditions (Supplementary Figure 4) since the intensity level of identified bait protein in different growth conditions is similar. Moreover, each of the queried replication proteins forms a unique constellation of contacts with the rest of the proteome with only small overlaps between baits (Fig. 11).

### Data reliability

Our data confirmed previously found, well-known interactions between complex components of several replication proteins, namely these formed by DNA polymerase III subunits, ribonucleotide reductase complex or between Hda and β sliding clamp of DNA polymerase III^14,34,35^. These are stable complexes that were isolated under all tested conditions. Our results also recapitulated the interactions described previously as spatiotemporally regulated during the cell cycle (DnaB-DnaC, DiaA-DnaA, HolD-Ssb, topoisomerase III-HolD)^14,36,37^ or performing special function, *i.e*. replication through highly transcribed regions (DnaB-Rep)^38^. These results confirm that the approach we used accurately identifies different types of complexes formed by the selected replication proteins.

## Usage Notes

### Navigating and visualizing the data

The goal of protein-protein interaction screens is often to determine which cellular processes are connected between each other. To test that, GO enrichment analysis are often performed. However, they are usually used to make specific conclusions rather than to organize the data. Here, we manually classified identified prey proteins into 10 functional categories (Table 5) to make navigating the data easier and let the user focus only on particular protein subgroups or notice the functional connections between replication and other processes, which can be an initial step for further functional studies. We are perfectly aware that such a classification is in many cases arbitral and might not reflect all the functions of proteins involved in different cellular processes (*i. e*. moonlighting enzymes), but anyway it may be useful when looking at the data from a wider perspective.

**Table 5:** Custom functional categories of prey proteins used in this study.

protein functional category	description
UNCHARACTERIZED/ORPHAN PROTEINS	proteins of undefined GO terms or uncharacterized biological function (experimentally)/orphan proteins
TRANSCRIPTION CONTROL PROTEINS	RNA polymerase subunits and transcription factors/transcription factors
RNA PROCESSING PROTEINS	RNA processing proteins (RNAses, proteins involved in RNA metabolism and modification, degradosome
RIBOSOMAL & TRANSLATION PROTEINS	ribosome biogenesis, ribosome subunits proteins, aa-tRNA ligases, translation proteins/ribosomal and translation proteins, rRNA/tRNA modifying proteins
METABOLIC & TRANSPORT PROTEINS	metabolic enzymes, transport proteins, secretion proteins, post-translational modification enzymes
CELL ENVELOPE PROTEINS	cell envelope proteins (proteins involved in synthesis/transport of cell membrane/cell wall components, membrane proteins)
DNA ARCHITECTURE/STRUCTURE PROTEINS	DNA binding proteins (not connected with transcription or not characterized), topoisomerases
DNA REPLICATION PROTEINS	DNA polymerase subunits, replication regulatory proteins, ssb protein
CHROMOSOME SEGREGATION & CELL DIVISION PROTEINS	FtsZ ring assembly proteins, FtsZ positioning system, chromosome segregation proteins
STRESS & STARVATION RESPONSE PROTEINS	DNA recombination, DNA recombinational repair, DNA repair, prophage integration, chaperone proteins, stres response, heat/cold/pH/starvation response proteins, DNA damage response, stringent response, SOS reponse etc.

Apart from uploading the datafiles containing protein lists, we visualized our data as PINs (Supplementary Figure 5A-H). Basically, manually made functional categories were used to arrange the PIN layout. Proteins belonging to each category were given different node colour as presented on the example network in Supplementary Figure 6; significantly enriched preys were distinguished from preys absent in both controls by node shape. Each of three tested growth conditions has different edge colour, in case of quantitatively processed data the edge width reflects the fold enrichment value with control 2 (heterologous SPA-tagged protein). Thus, by looking at the network, the user will find numerous information about the protein-protein interactions. PINs of quantitatively and qualitatively processed data are deposited in the NDEX project (links below) in the interactive form, with every parameter easily accessible:

https://www.ndexbio.org/#/networkset/4555289d-18d6-11ed-ac45-0ac135e8bacf?accesskey=2986992b747495b613f833709fe92d5dce65cdf9c731b0b0a56af07ef01e023e

https://www.ndexbio.org/#/networkset/53cc0577-196e-11ed-ac45-0ac135e8bacf?accesskey=d136944dff0a03b5effb16643f21d07ab33d8d3e4bfad1f16a190d3d13959144

### Reusing the data

In this work we present AP-MS dataset processed in two distinct ways. According to the established criteria, different proteins might be considered as specific interactants. Thus, one can reprocessed the data using different criteria. For example, in the case of qualitative data processing protein hits that appeared in at least two out of three replicates might also be considered. In the case of quantitative data processing, in turn, less restrictive (*i. e*. 0.05) p-value or different fold enrichment threshold may be chosen. Obviously, the user can also take the raw MaxQuant data^29^ and process it in a totally different way.

The double-control system design as well as processing strategies presented in this work can be used with the data generated from other C-terminal SPA-tag *E. coli* strains of different genetic backgrounds as well as in other AP-MS datasets identified using MaxQuant environment. Complex data processing presented here may constitute a cheaper and easier alternative to quantitative AP-MS methods using isotope labelling.

### Supplementary information

Supplementary information

## Data Availability

The code used to assess significance of the interactions was deposited under the following link: https://github.com/MaximeCarlier12/Interactome_proteins_ecoli. The MIT license is available at: https://github.com/MaximeCarlier12/Interactome_proteins_ecoli/blob/master/LICENSE.
