# Supplementary material for: Interaction networks of Escherichia coli replication proteins under different bacterial growth conditions
Source: Sci Data. 2023 Nov 10;10:788. doi: 10.1038/s41597-023-02710-1 (PMC10638427; doi:10.1038/s41597-023-02710-1)
Supplement: Supplementary file 1 — Supplementary information [file 41597_2023_2710_MOESM1_ESM.pdf]

Interaction networks of *Escherichia coli* replication proteins under different bacterial growth conditions

Supplementary information

Table of contents:

Supplementary Figure 1	Page 2
Supplementary Figure 2	Page 3
Supplementary Figure 3	Page 4-11
Supplementary Figure 4	Page 11
Supplementary Figure 5	Page 12-20
Supplementary Figure 6	Page 21
Supplementary Table 1 (provided as separate .xlsx file as well)	Page 22-23

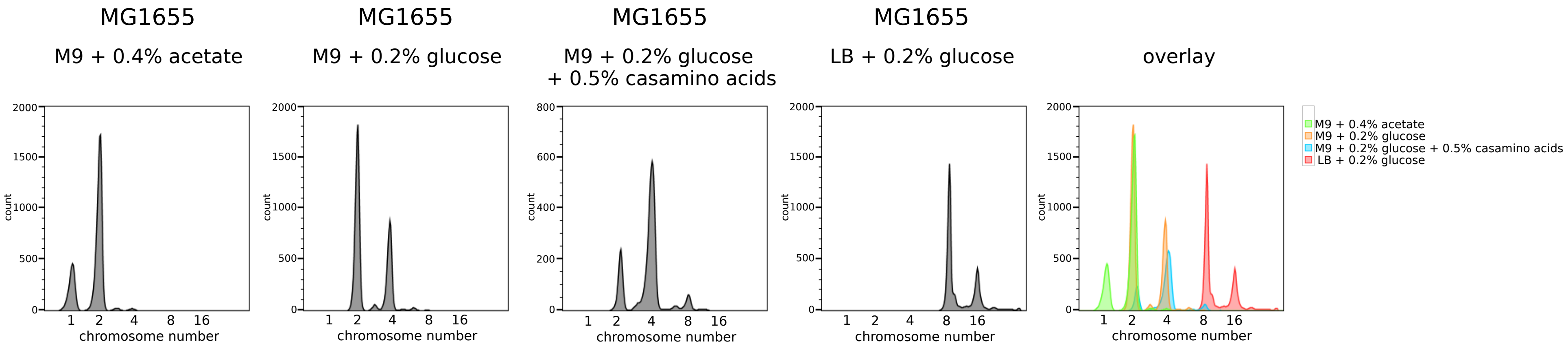

Supplementary figure 1

Flow cytometry histograms of MG1655 (genetic background) strain grown in 4 different growth conditions until early logarithmic growth phase (OD=0.15) and subjected to inhibition of new replication rounds as well as cell divisions. After completion of ongoing replication rounds the cells contain chromosome number equal to the number of origins present in the cell when inhibitors were added. Chromosomal DNA is then stained with a fluorescent dye and the DNA content is measured by flow cytometry. Cell populations contain particular chromosome number, depending on the growth rate.

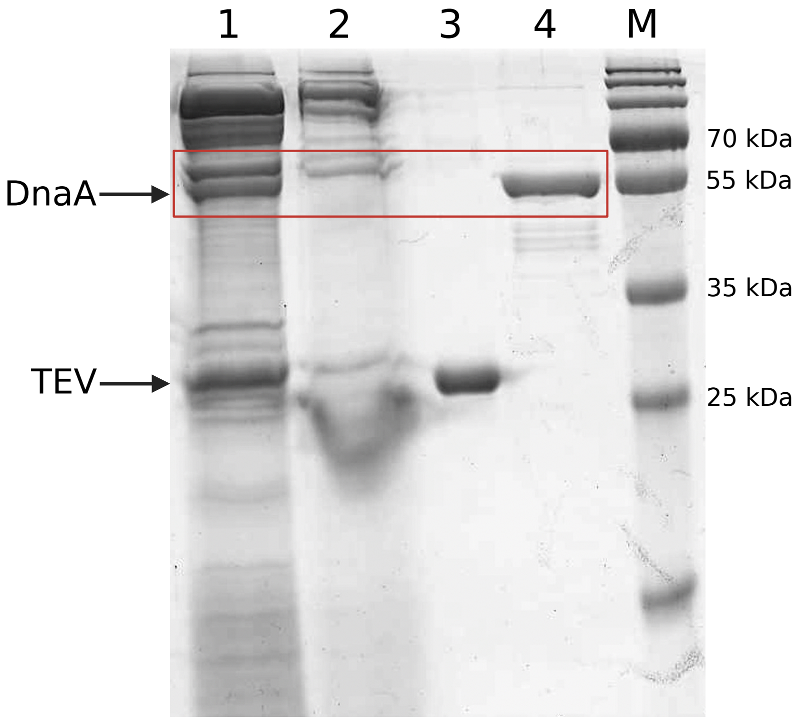

1 - elution sample after TEV cleavage from anti-FLAG affinity resin
2 - anti-FLAG affinity resin after TEV cleavage
3 - purified TEV
4 - purified DnaA
M - PageRuler™ Plus Prestained Protein Ladder

Supplementary figure 2

A test of TEV cleavage efficiency of the SPA-tagged DnaA protein bound to anti-FLAG resin. Samples were resolved in 10% SDS-PAGE gel, lane content was depicted in the picture. SPA-tagged DnaA was isolated from the respective strain according to the described protocol.

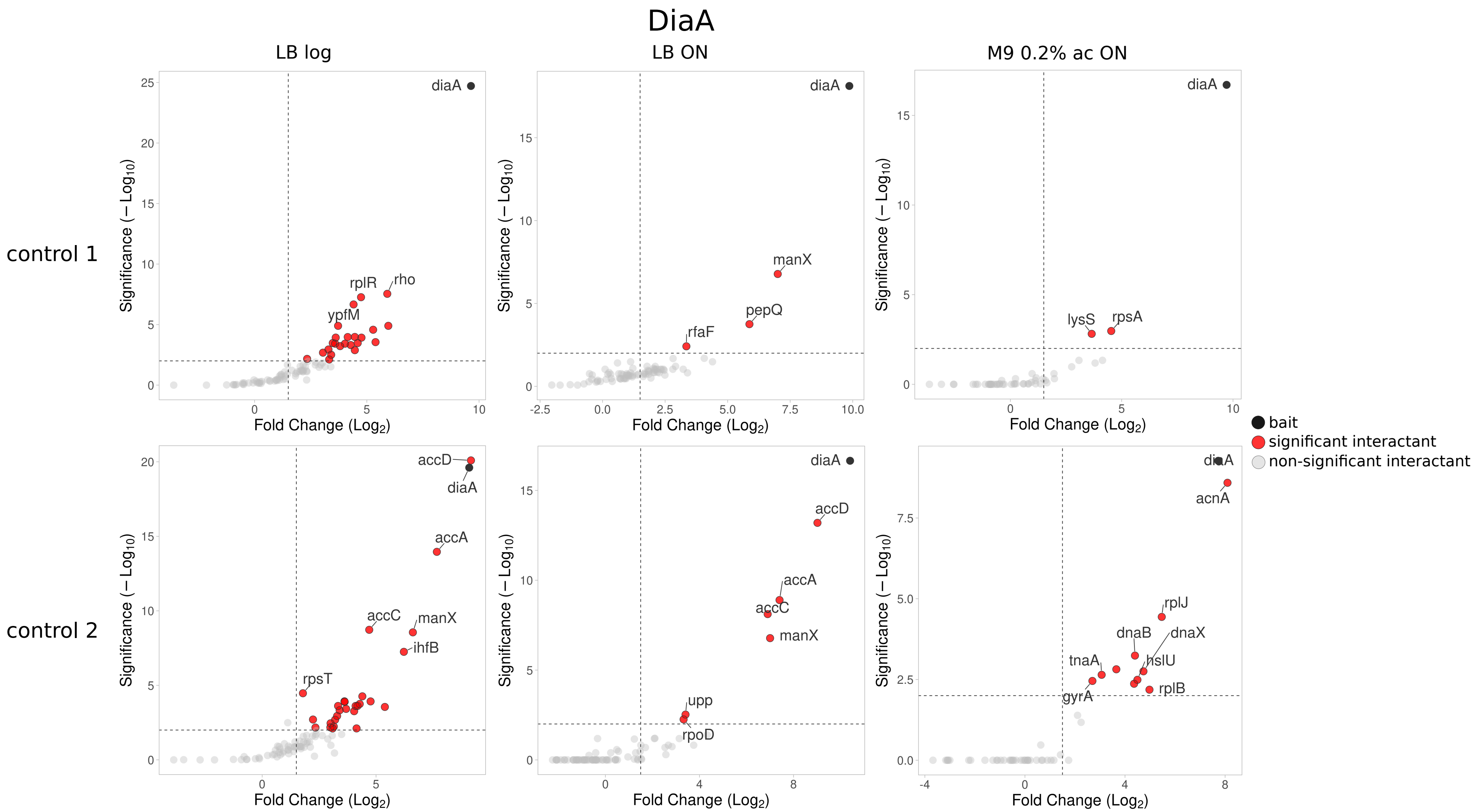

Supplementary figure 3A

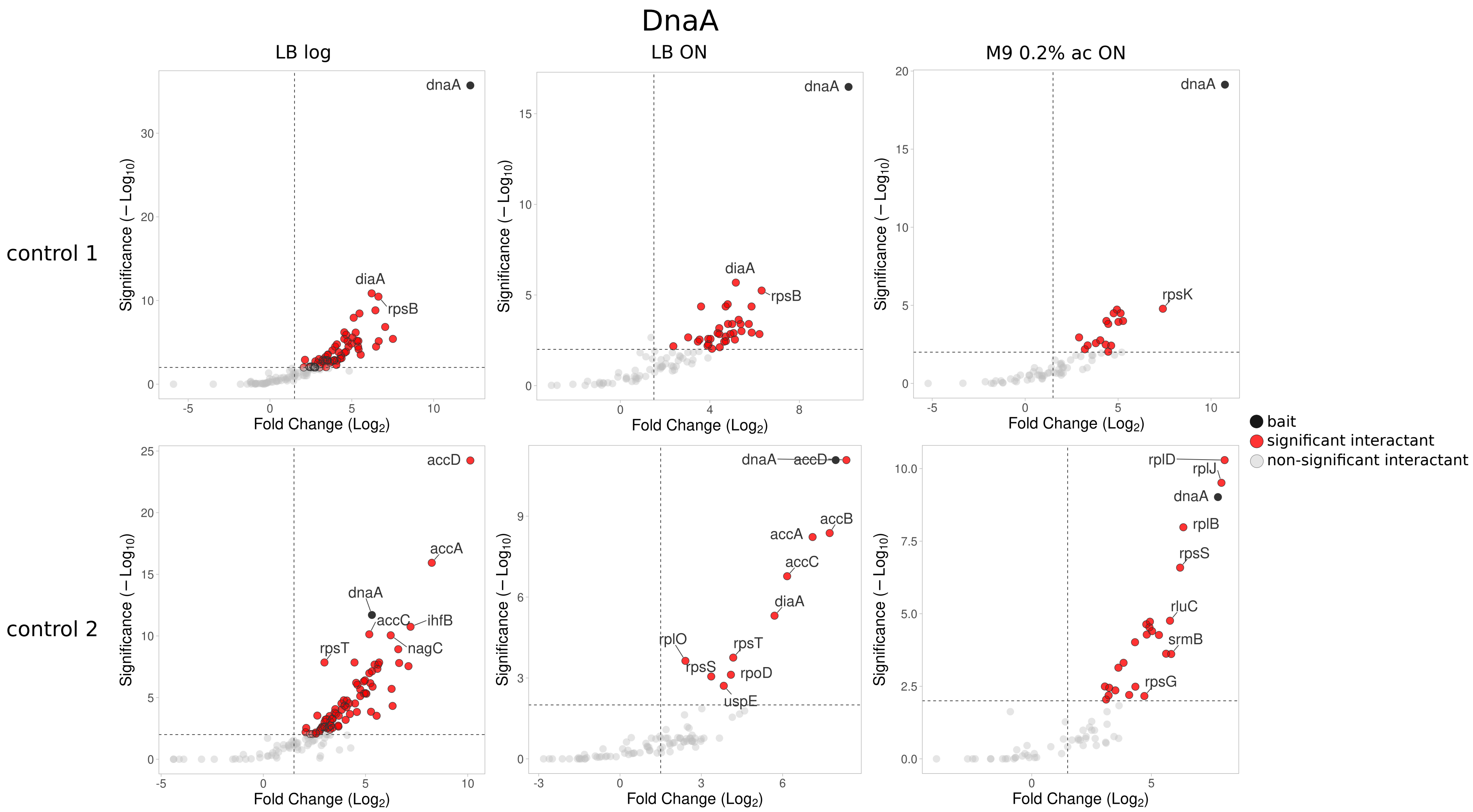

Supplementary figure 3B

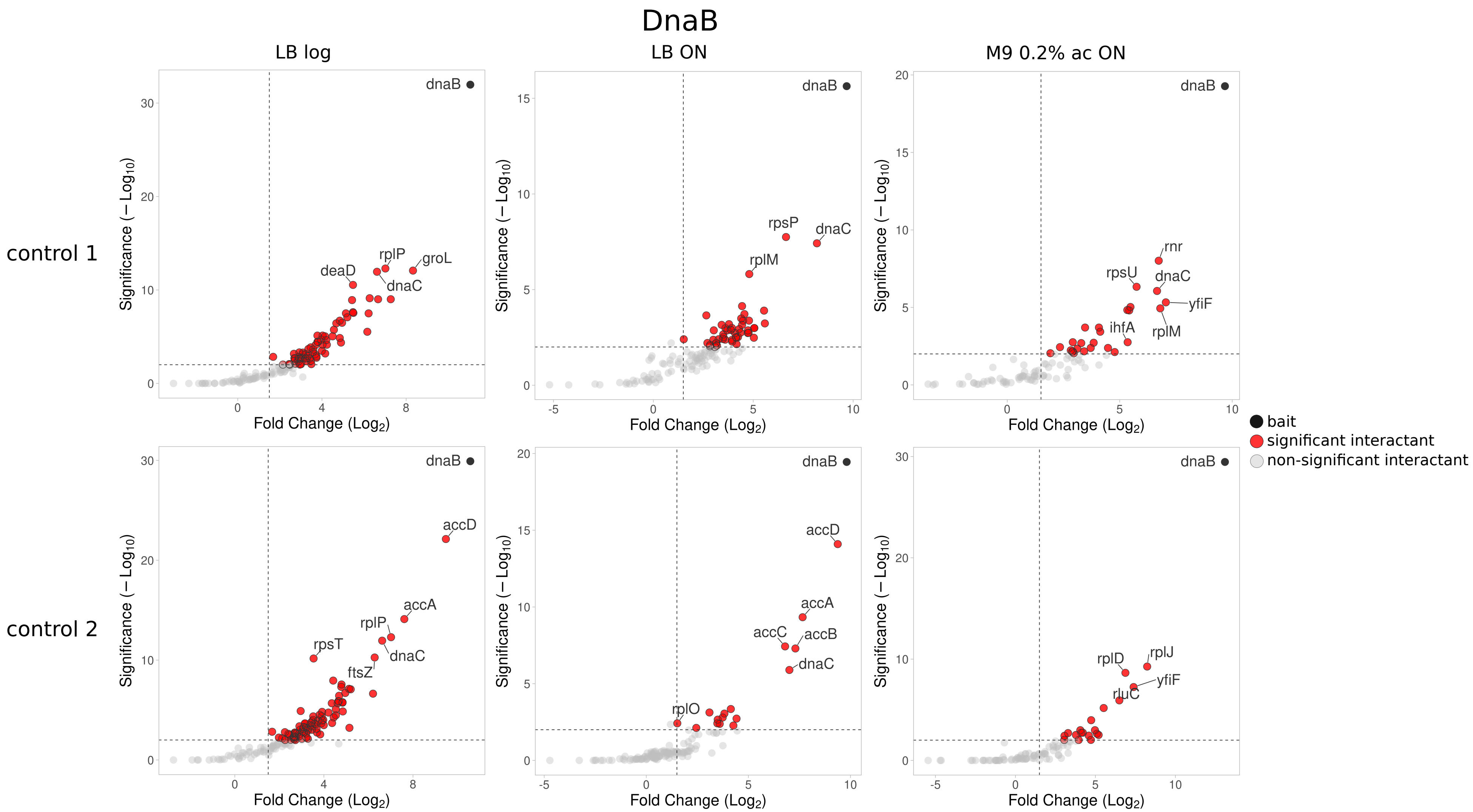

Supplementary figure 3C

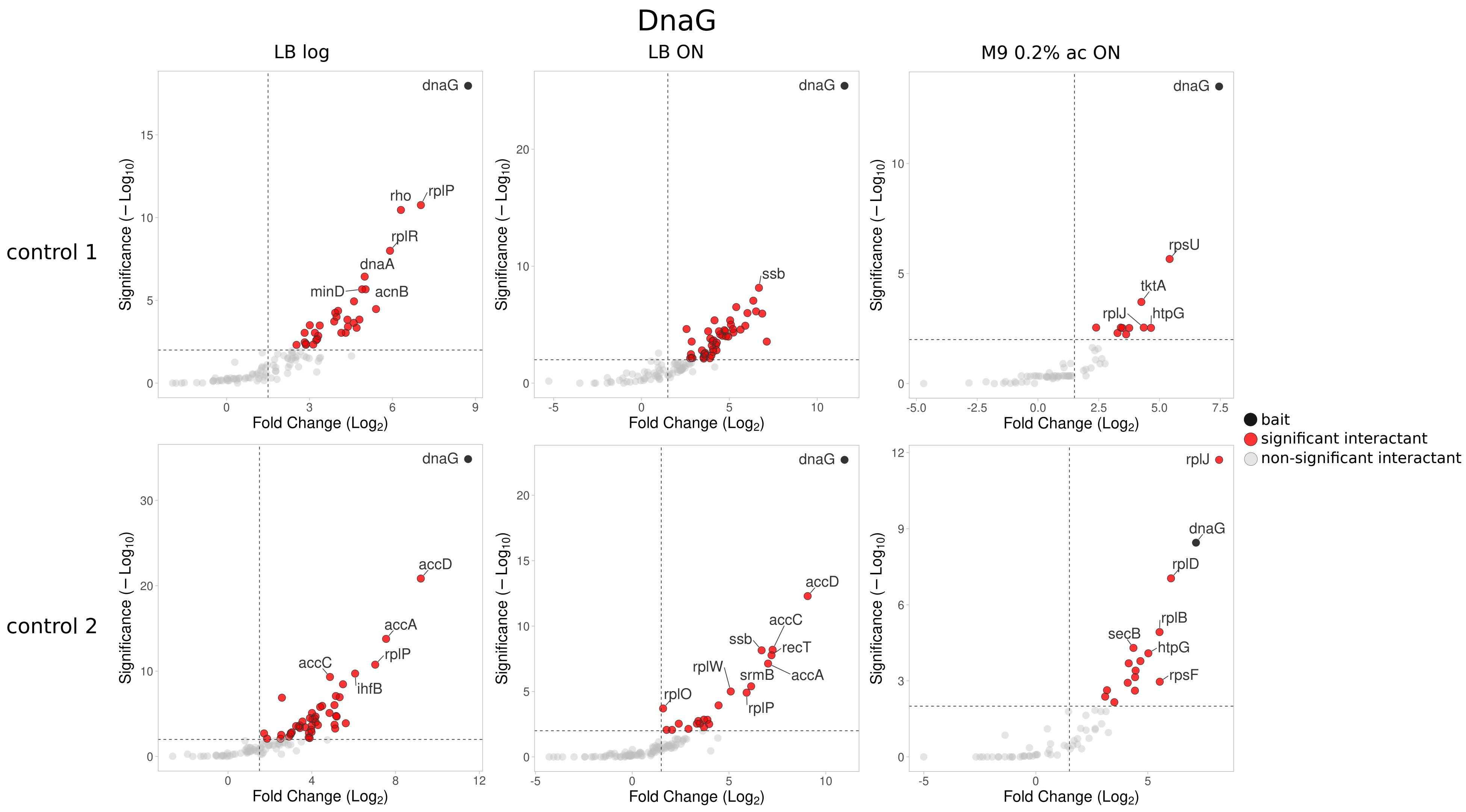

Supplementary figure 3D

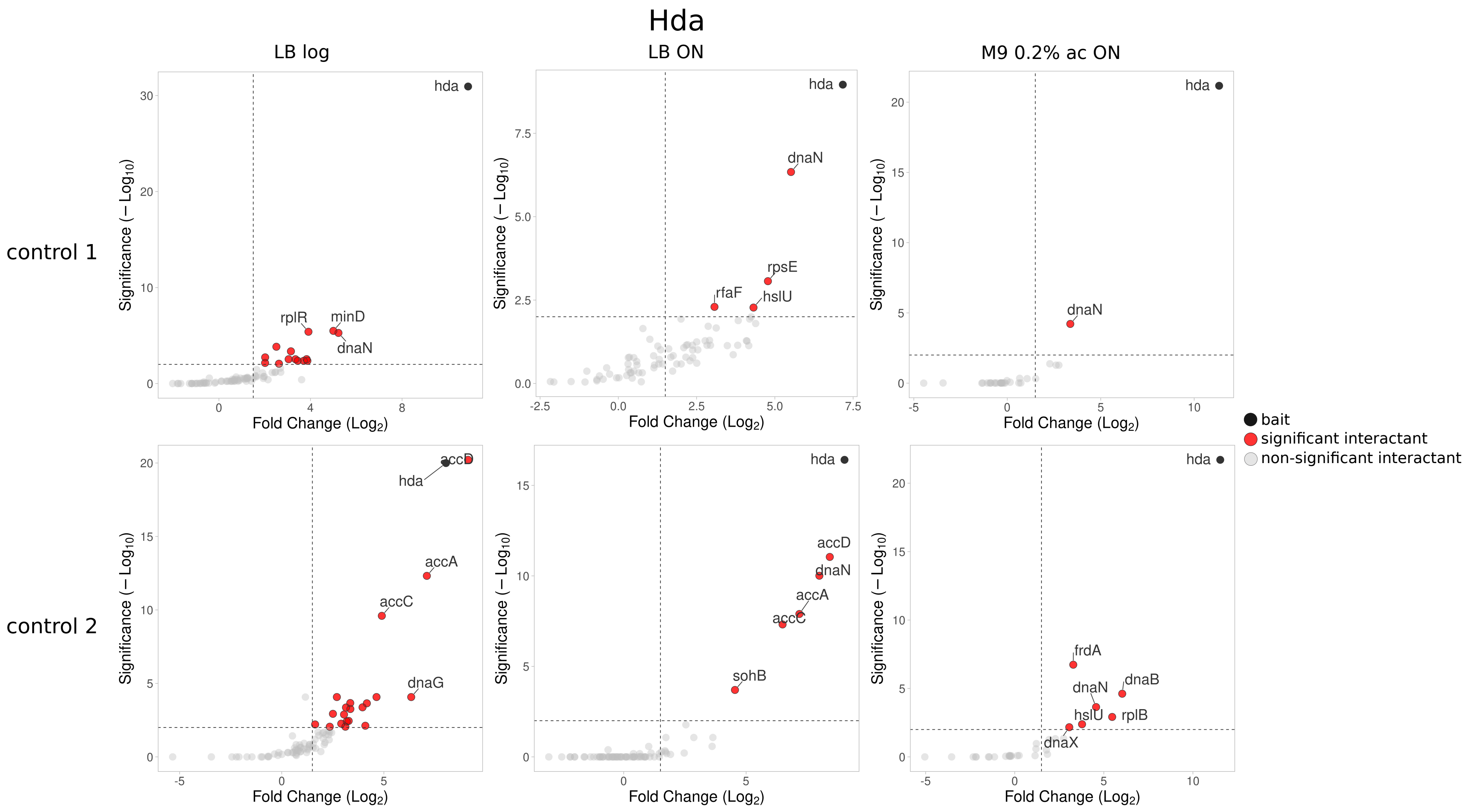

Supplementary figure 3E

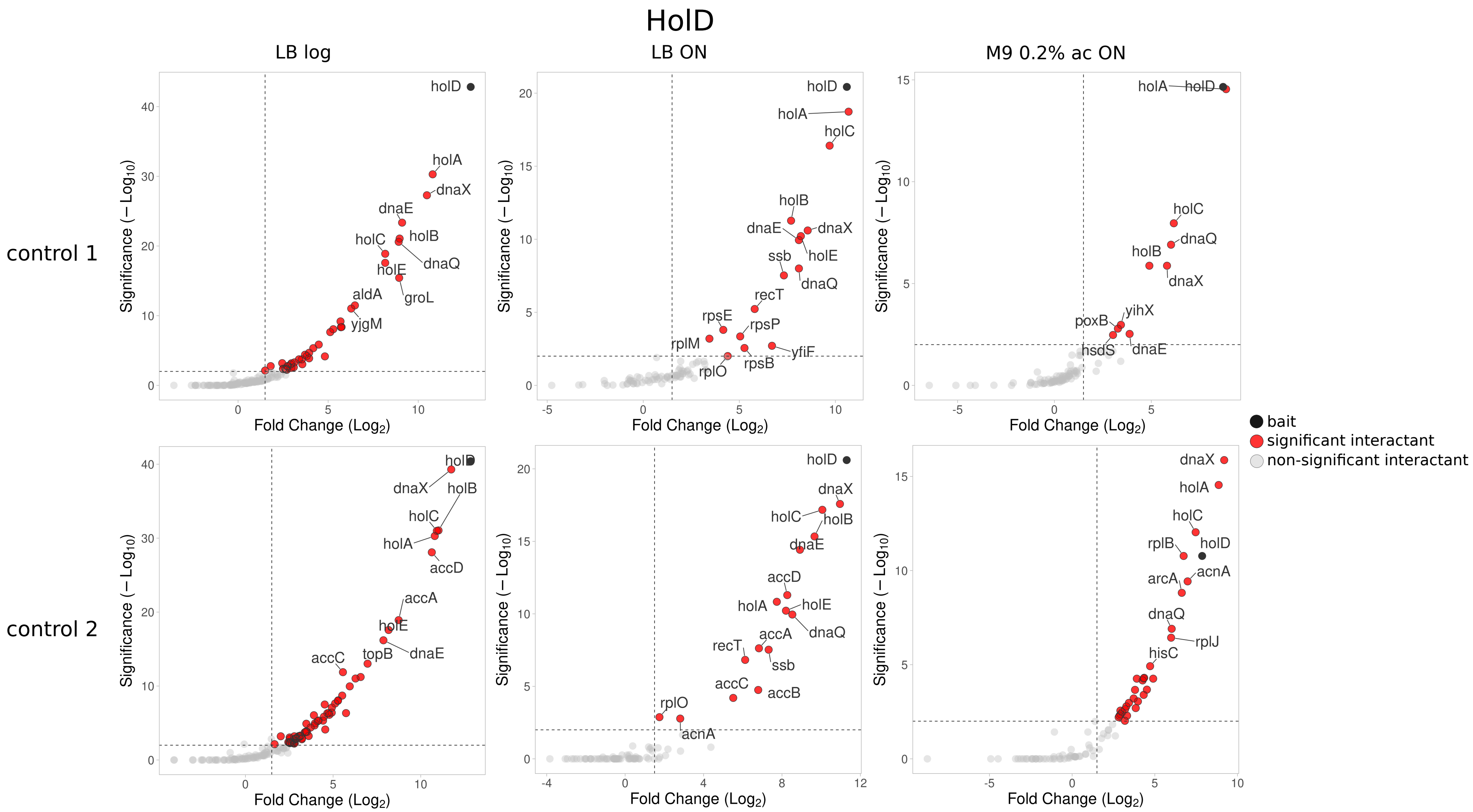

Supplementary figure 3F

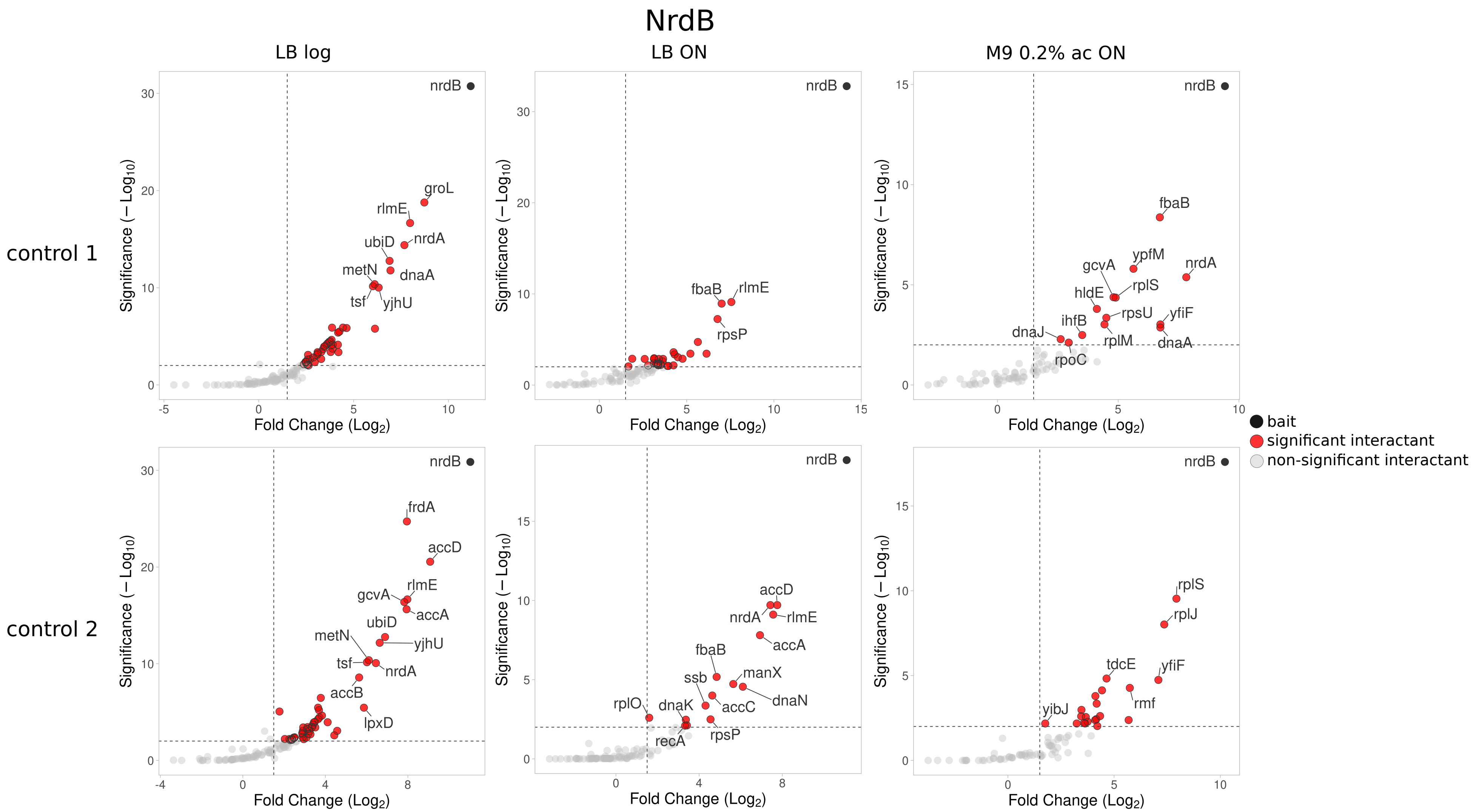

Supplementary figure 3G

Supplementary figure 3
Volcano plots depicting enrichment and statistical significance of uncovered interactions for various bait proteins:

A) DiaA
B) DnaA
C) DnaB
D) DnaG
E) Hda
F) HolD
G) NrdB

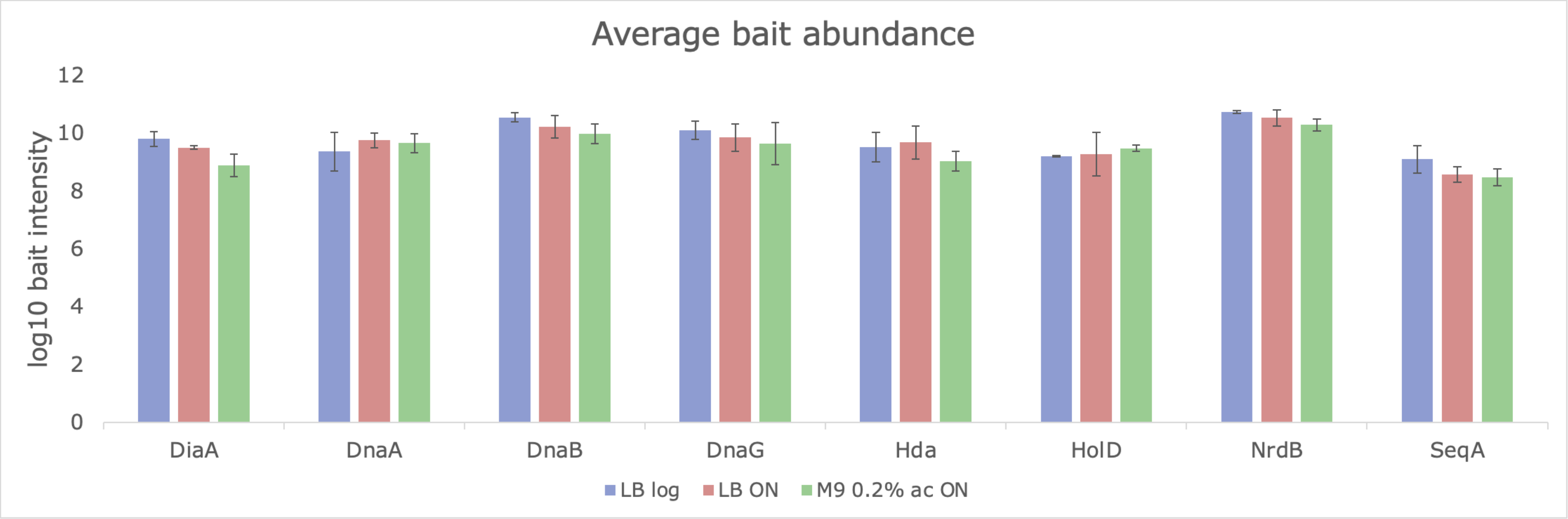

Supplementary figure 4
Average bait abundance shown as log10 of bait intensity value across different growth conditions of experimental samples.

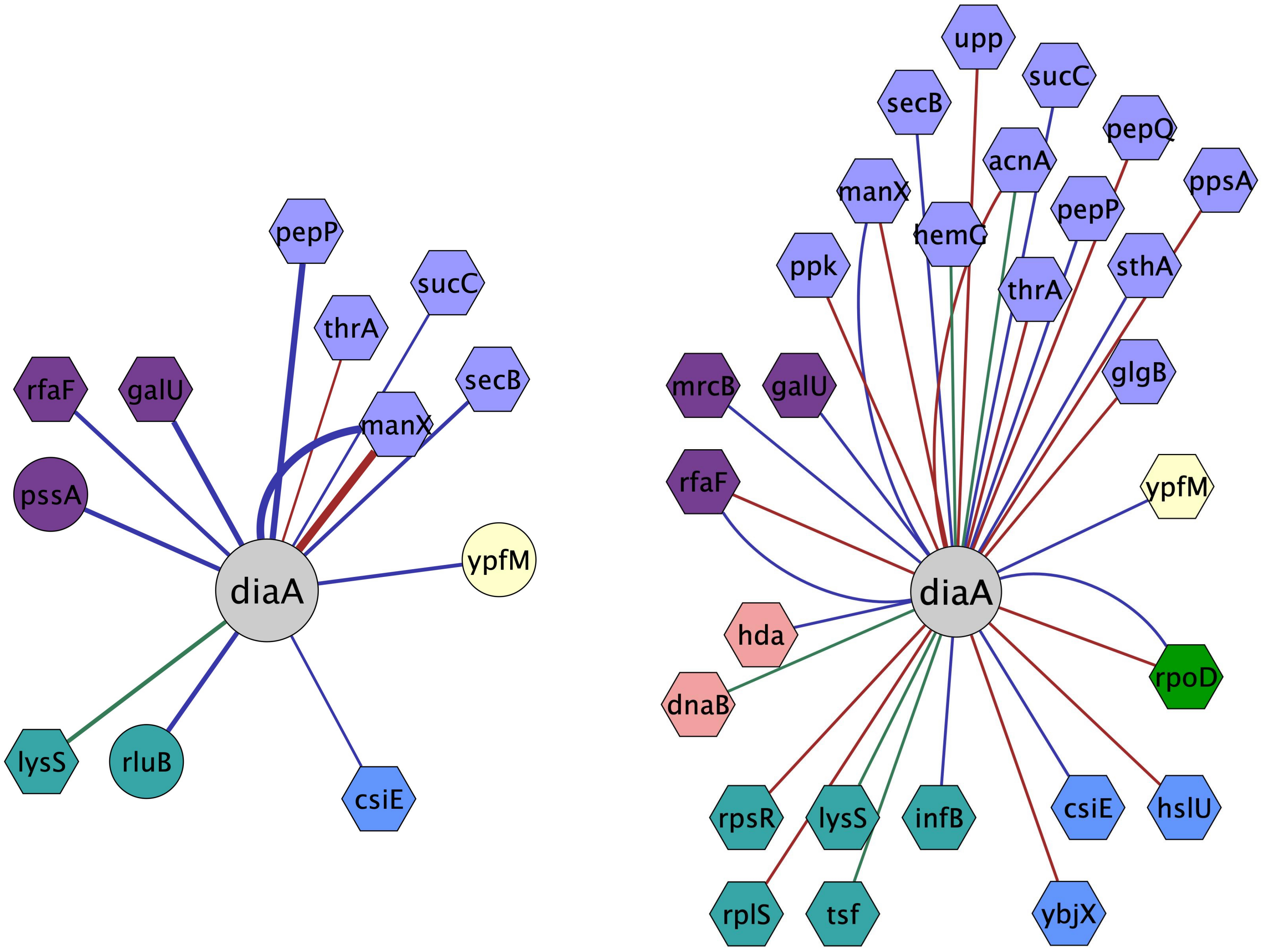

Supplementary figure 5A

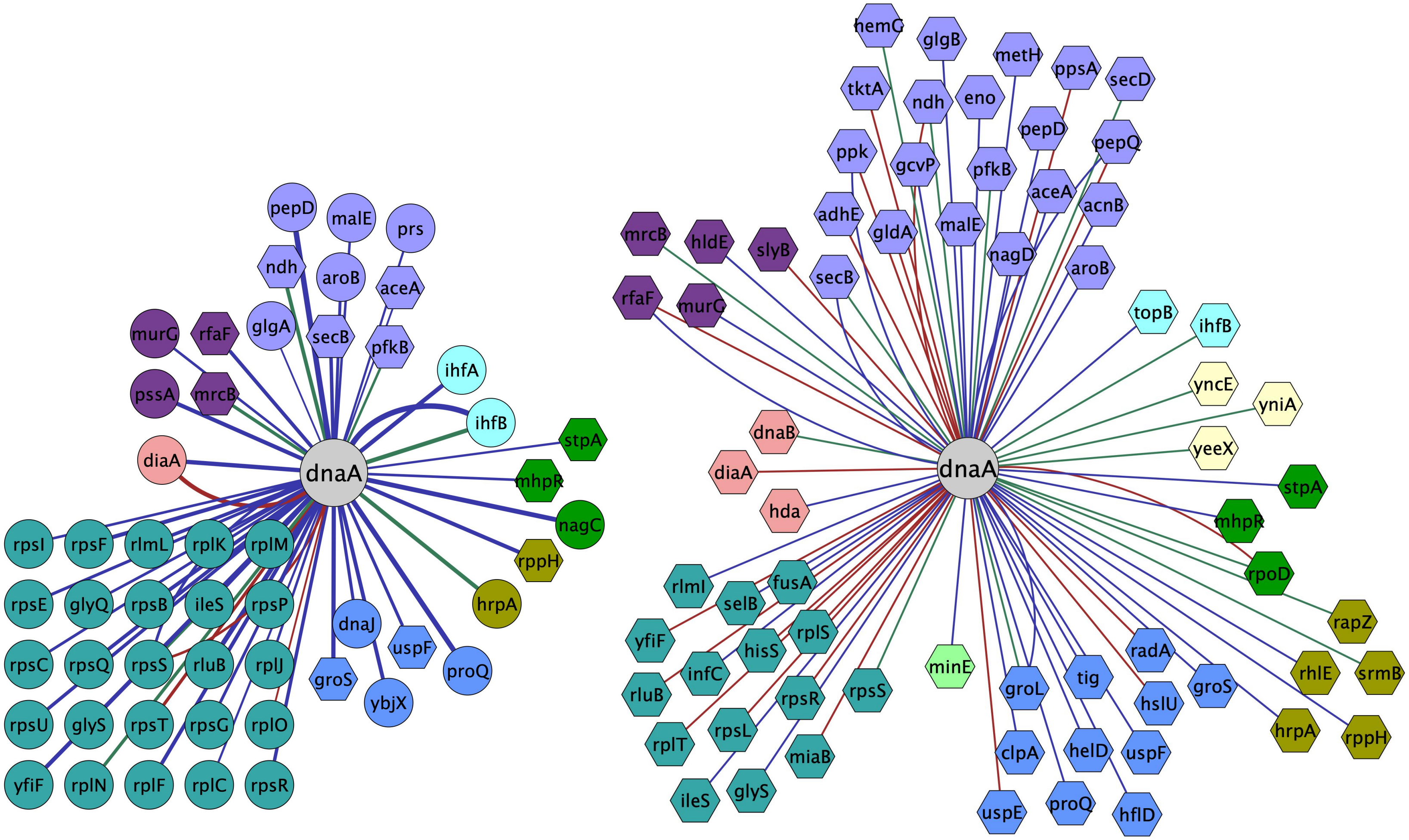

Supplementary figure 5B

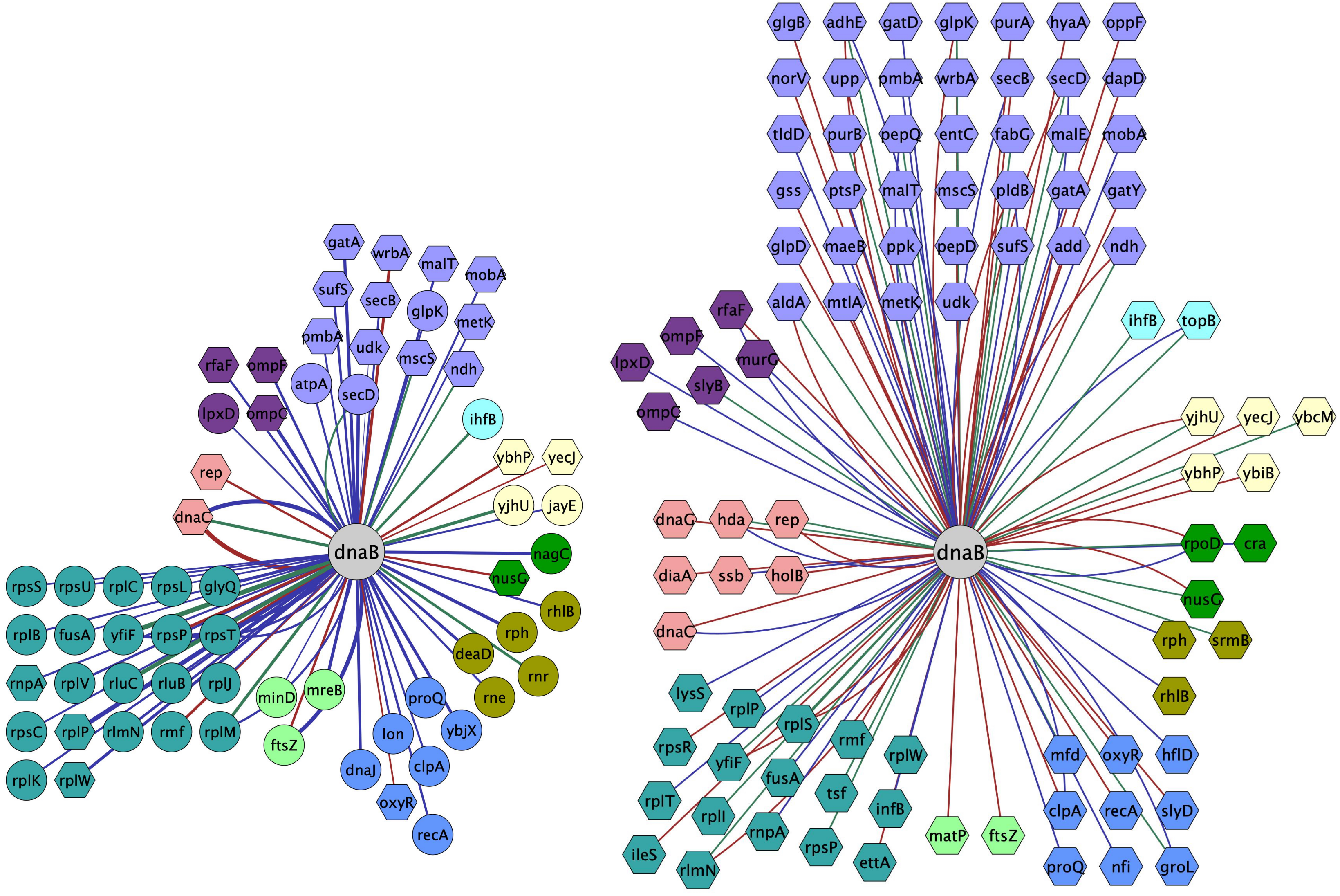

Supplementary figure 5C

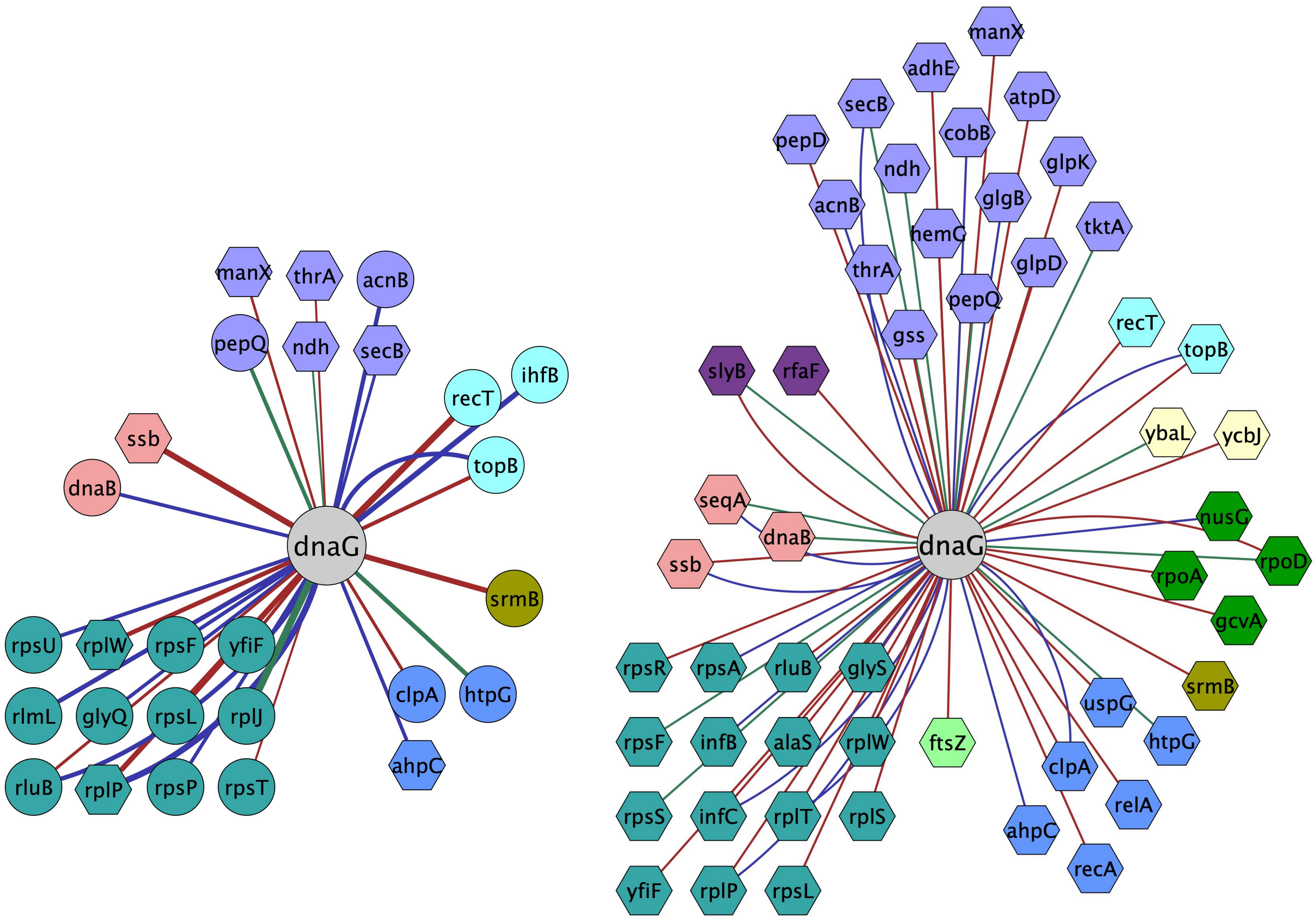

Supplementary figure 5D

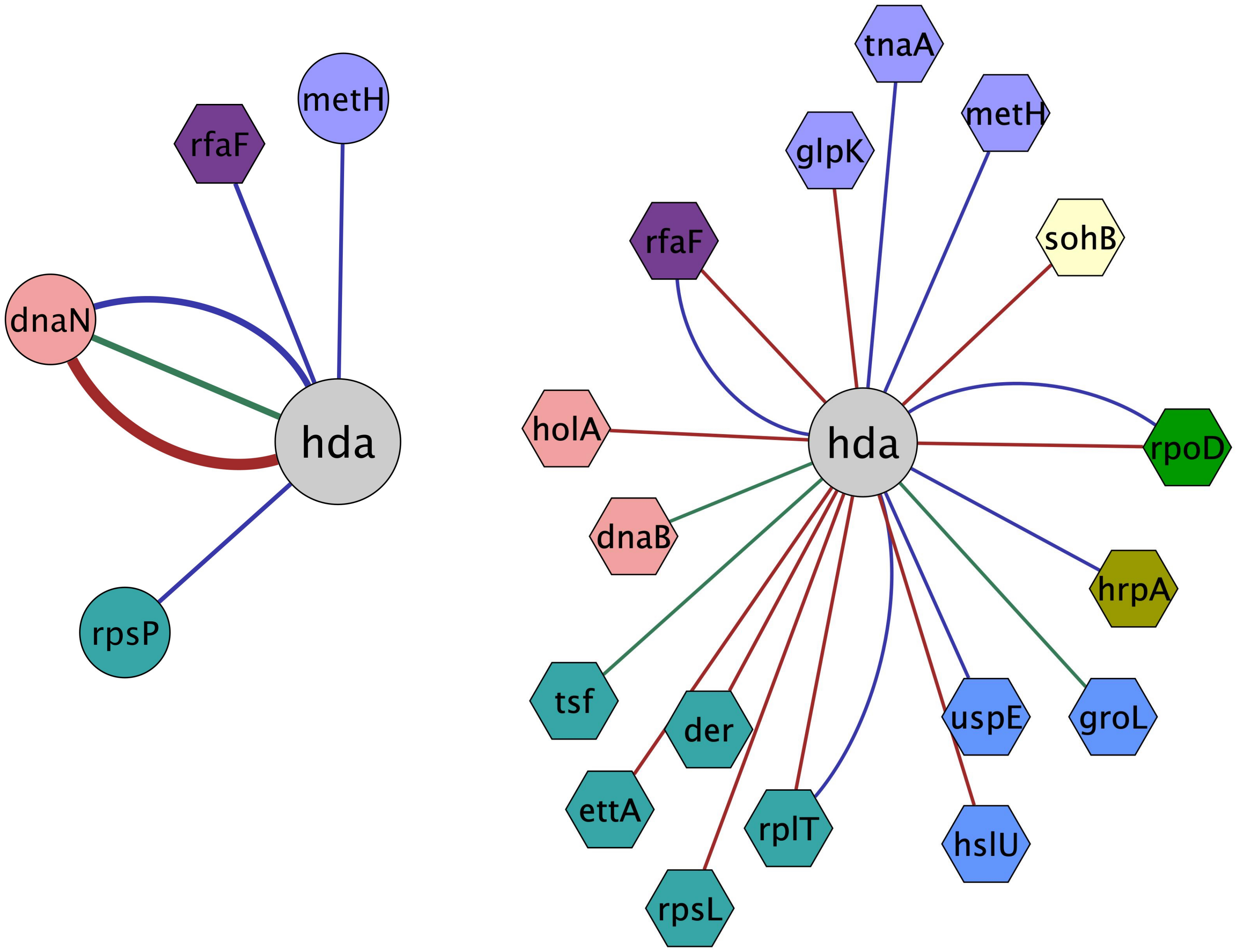

Supplementary figure 5E

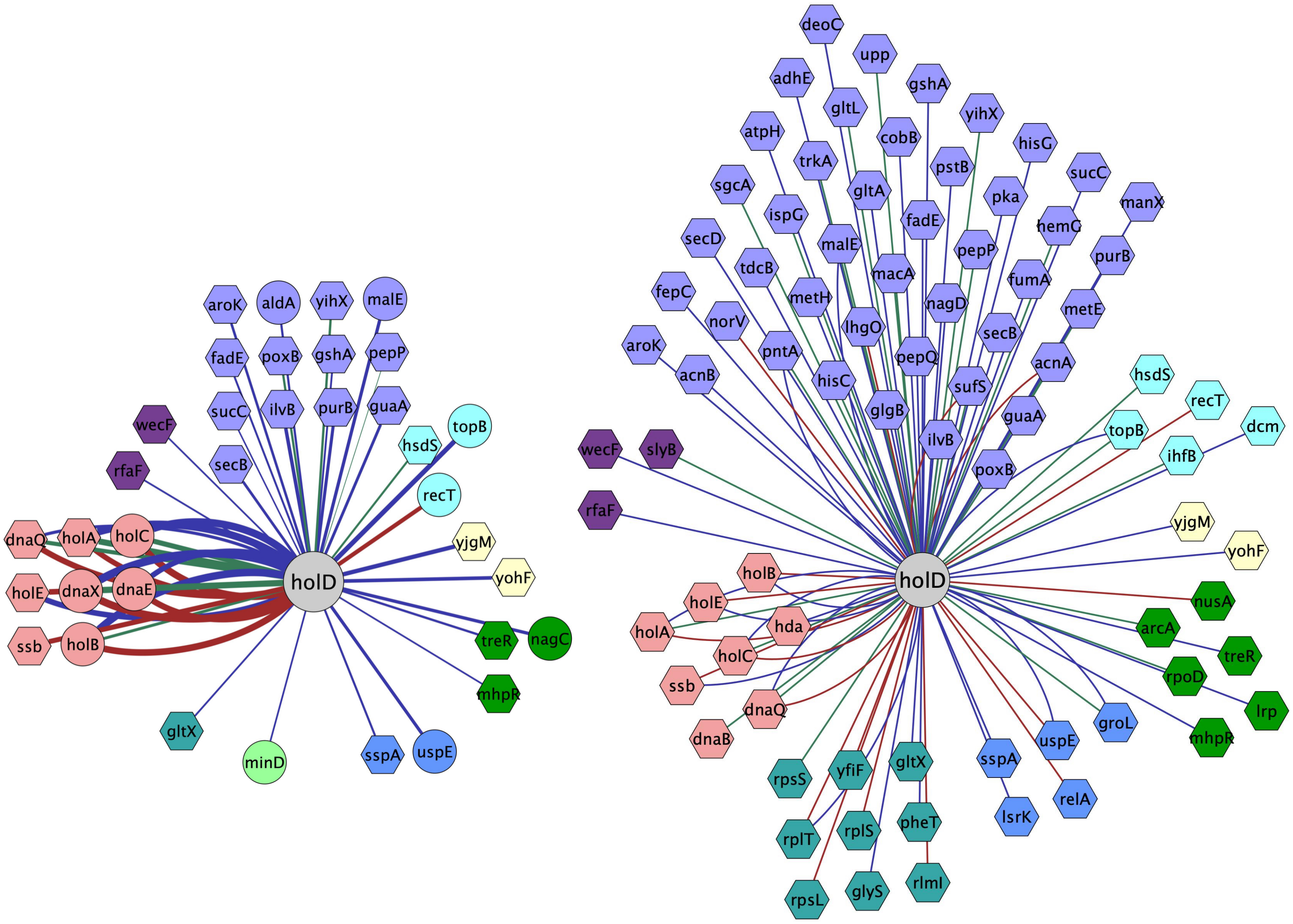

Supplementary figure 5F

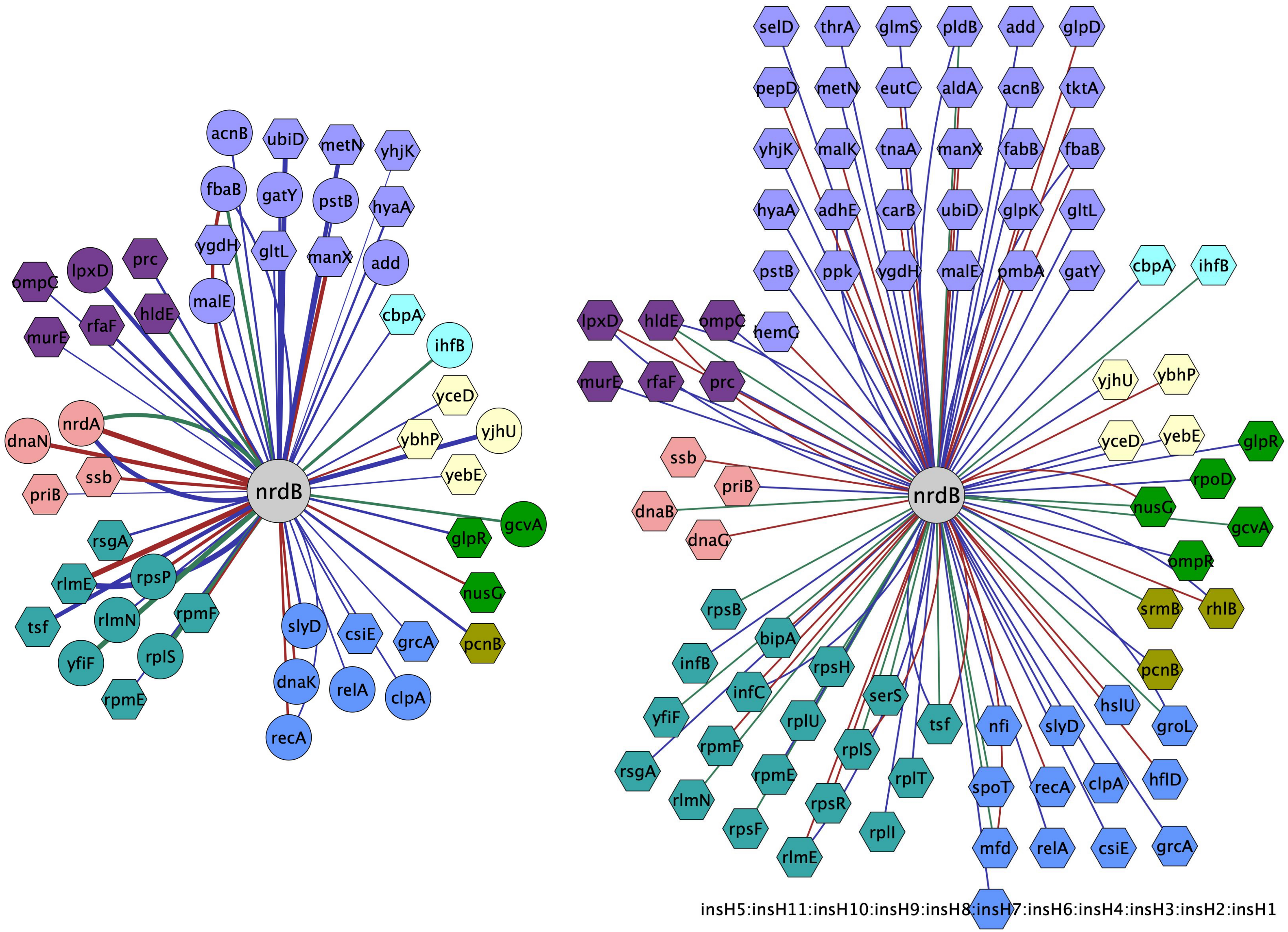

Supplementary figure 5G

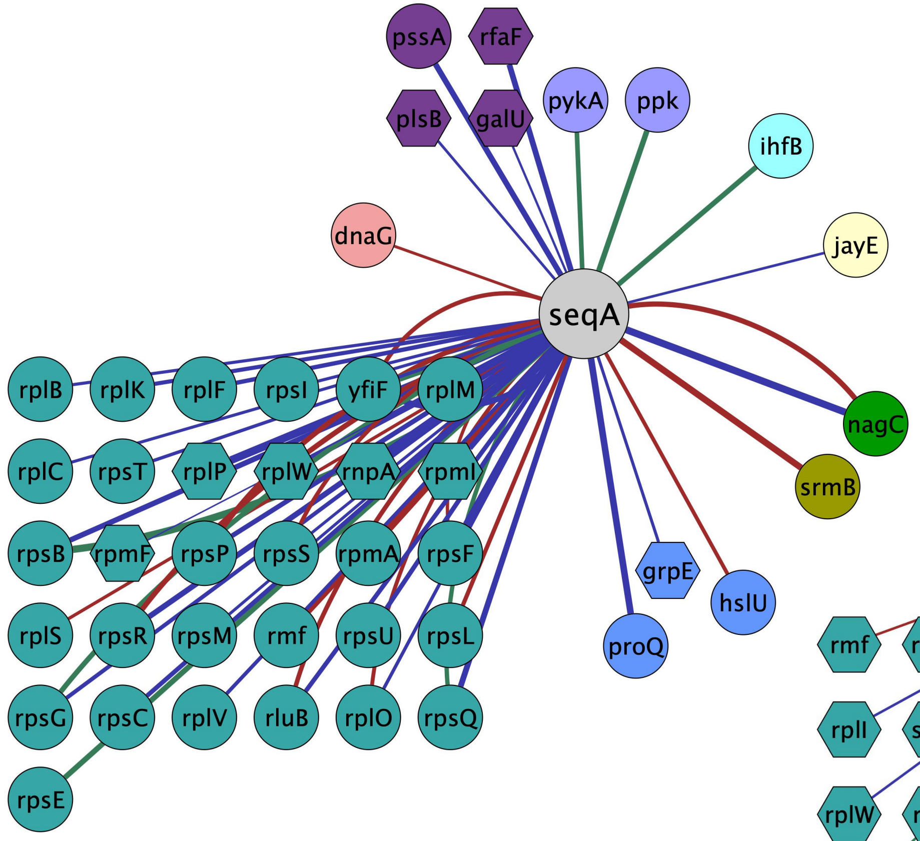

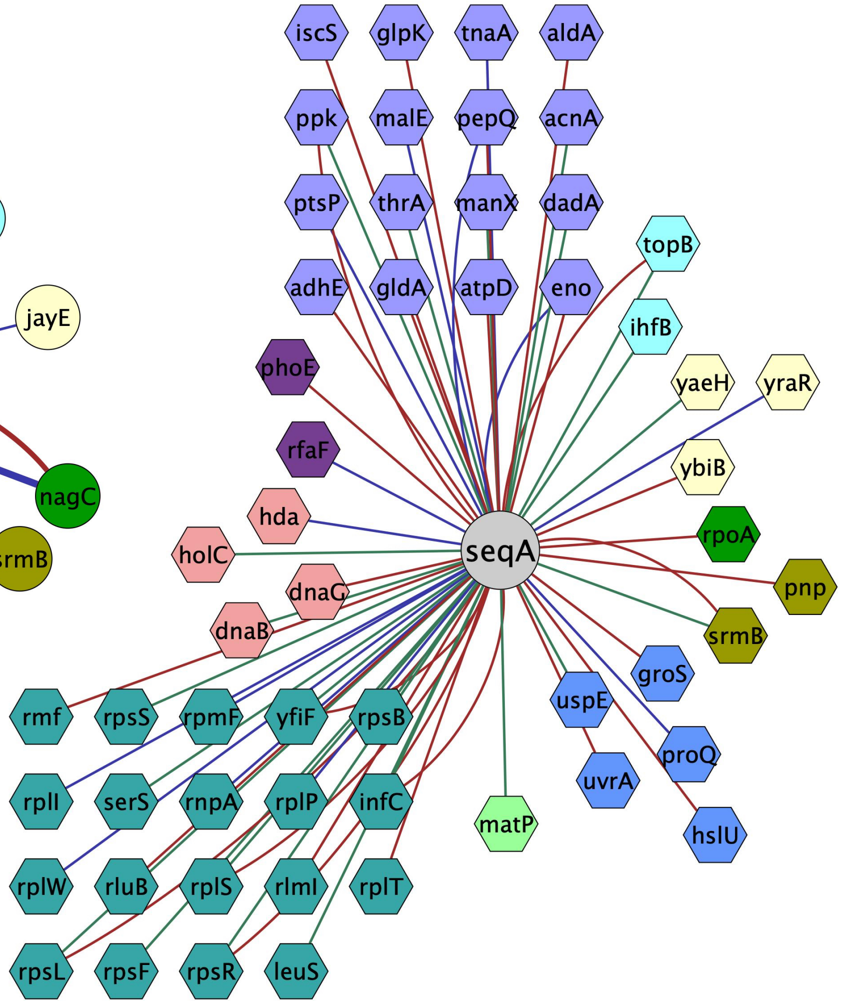

Supplementary figure 5H

Supplementary figure 5

A) PINs for DiaA - data processed quantitatively (left) and qualitatively (right).
B) PINs for DnaA - data processed quantitatively (left) and qualitatively (right).
C) PINs for DnaB - data processed quantitatively (left) and qualitatively (right).
D) PINs for DnaG - data processed quantitatively (left) and qualitatively (right).
E) PINs for Hda - data processed quantitatively (left) and qualitatively (right).
F) PINs for HolD - data processed quantitatively (left) and qualitatively (right).
G) PINs for NrdB - data processed quantitatively (left) and qualitatively (right).
H) PINs for SeqA - data processed quantitatively (left) and qualitatively (right).

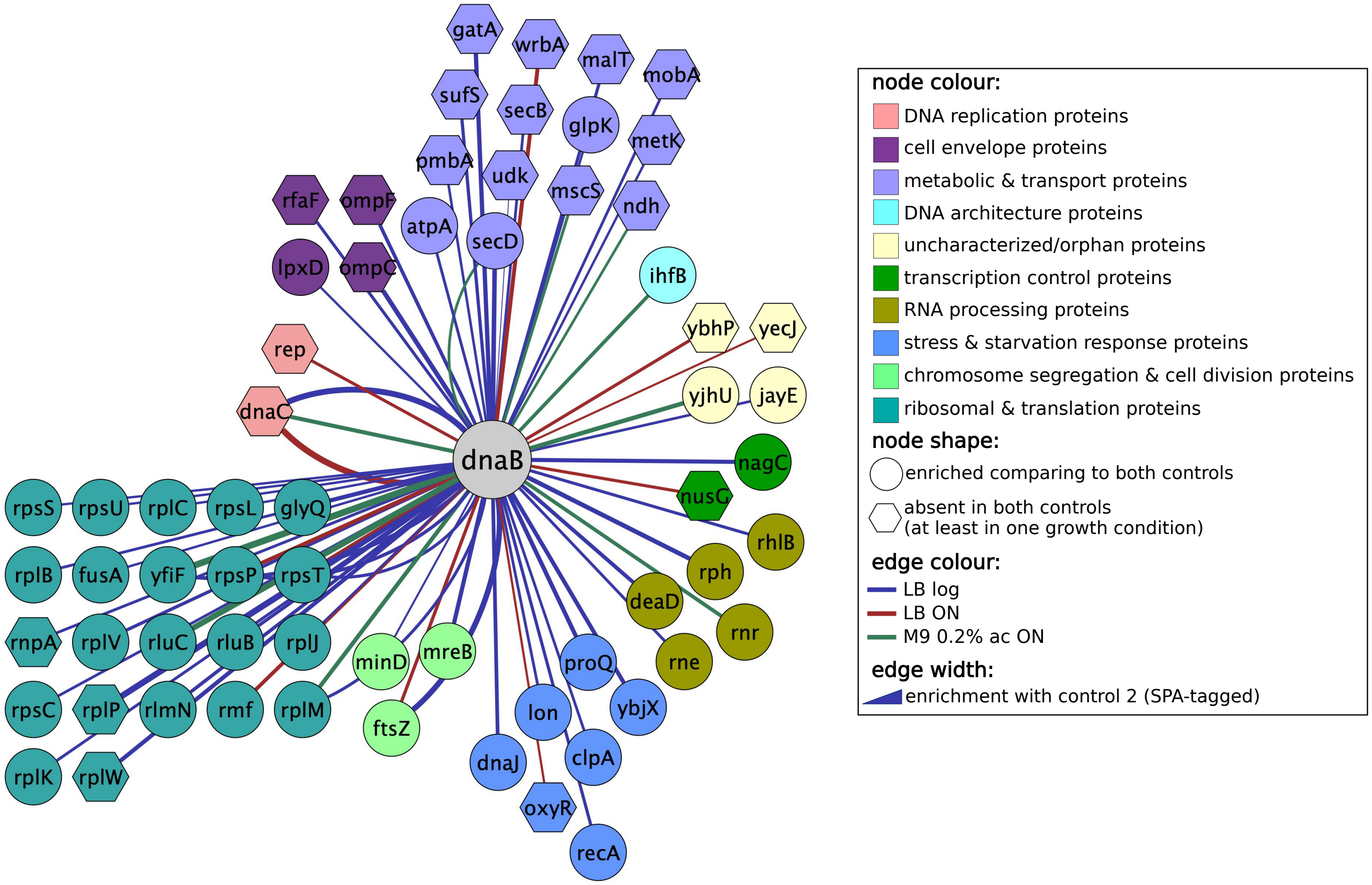

Supplementary figure 6
Example PIN along with the legend explaining how to read different nodes' and edges' parameters.

Supplementary Table 1
MaxQuant data sample coding along with corresponding raw MS datafiles' names

	MaxQuant data sample coding			raw MS data full file name in PRIDE		
sample type (experimental(E)/control(C)): bait protein	growth condition			growth condition		
	LB log	LB O/N	M9 0.2% ac O/N	LB log	LB O/N	M9 0.2% ac O/N
E:**DiaA**	E1; I4; X6	I5; P1; X7	I6; T3; X5	70408550glin_E1.raw; 709011793glin_I4.raw; 008131321glin_X6.raw	709011794glin_I5.raw; 908161515glin_P1.raw; 008131322glin_X7.raw	709011795glin_I6.raw; 911302356glin_T3.raw; 008131320glin_X5.raw
E:**DnaA**	F2; U6; U7	F1; P2; X12	E3; I7; X11	70524852glin_F2.raw; 912142457glin_U6.raw; 912142458glin_U7.raw	70524851glin_F1.raw; 908161516glin_P2.raw; 008131327glin_X12.raw	70408552glin_E3.raw; 709011796glin_I7.raw; 008131326glin_X11.raw
E:**DnaB**	G4; W3; W4	G6; W5; W6	G2; W1; W2	706281047glin_G4.raw; 00319348glin_W3.raw; 00319349glin_W4.raw	706281049glin_G6.raw; 00319350glin_W5.raw; 00319351glin_W6.raw	706281045glin_G2.raw; 00319346glin_W1.raw; 00319347glin_W2.raw
E:**DnaG**	H1; J5; U9	C7; F6; H2	F7; H3; X15	706281185glin_H1.raw; 710192290glin_J_5.raw; 912142460glin_U9.raw	611072350glin_C_7.raw; 70524856glin_F6.raw; 706281186glin_H2.raw	70524857glin_F7.raw; 706281187glin_H3.raw; 008131330glin_X15.raw
E:**Hda**	C5; U8; X9	C6; P3; X10	I3; T4; X8	611072348glin_C_5.raw; 912142459glin_U8.raw; 008131324glin_X9.raw	611072349glin_C_6.raw; 908161517glin_P3.raw; 008131325glin_X10.raw	709011792glin_I3.raw; 911302357glin_T4.raw; 008131323glin_X8.raw
E:**HolD**	U1; U2; U3	F8; U11; X4	X1; X2; X3	912142452glin_U1.raw; 912142453glin_U2.raw; 912142454glin_U3.raw	70524858glin_F8.raw; 912142462glin_U11.raw; 008131319glin_X4.raw	008131316glin_X1.raw; 008131317glin_X2.raw; 008131318glin_X3.raw

E:**NrdB**	W7; W8; W9	F10; J4; U13	F9; J3; T10	00319352glin_W7.raw; 00319353glin_W8.raw; 00319354glin_W9.raw	70524860glin_F10.raw; 710192289glin_J_4.raw; 912142464glin_U13.raw	70524859glin_F9.raw; 710192288glin_J_3.raw; 911302363glin_T10.raw
E:**SeqA**	F4; X13; X14	F3; P4; U12	F5; T1; T2	70524854glin_F4.raw; 008131328glin_X13.raw; 008131329glin_X14.raw	70524853glin_F3.raw; 908161518glin_P4.raw; 912142463glin_U12.raw	70524855glin_F5.raw; 911302354glin_T1.raw; 911302355glin_T2.raw
C: MG1655 **(TYPE 1 CONTROL**)	T7; T8; T9	P5; U10; X17	T5; T6; X16	911302360glin_T7.raw; 911302361glin_T8.raw; 911302362glin_T9.raw	908161519glin_P5.raw; 912142461glin_U10.raw; 008131332glin_X17.raw	911302358glin_T5.raw; 911302359glin_T6.raw; 008131331glin_X16.raw
C:MG1655 (placI)mVenus-SPA-pUC19 **(TYPE 2 CONTROL)**	S1; S2; S3	O9; R4; R5	R1; R2; R3	910182130glin_S1.raw; 910182131glin_S2.raw; 910182132glin_S3.raw	901043208glin_O9.raw; 909131890glin_R4.raw; 909131891glin_R5.raw	909131887glin_R1.raw; 909131888glin_R2.raw; 909131889glin_R3.raw
